# The receptor of the colony-stimulating factor-1 (CSF-1R) is a novel prognostic factor and therapeutic target in follicular lymphoma

**DOI:** 10.1038/s41375-021-01201-9

**Published:** 2021-03-17

**Authors:** Juan Garcia Valero, Alba Matas-Céspedes, Fabián Arenas, Vanina Rodriguez, Joaquim Carreras, Neus Serrat, Martina Guerrero-Hernández, Anella Yahiaoui, Olga Balagué, Silvia Martin, Cristina Capdevila, Lluis Hernández, Laura Magnano, Alfredo Rivas-Delgado, Stacey Tannheimer, Maria C. Cid, Elías Campo, Armando López-Guillermo, Dolors Colomer, Patricia Pérez-Galán

**Affiliations:** 1 Department of Hematology-Oncology, IDIBAPS, Barcelona, Spain; 2 Centro de Investigación Biomédica en Red-Oncología (CIBERONC), Madrid, Spain; 3 Department of Pathology, Tokai University, School of Medicine, Isehara, Kanagawa Japan; 4 Gilead Sciences, Inc, Foster City, USA; 5 Hematopathology Unit, Pathology Department, Hospital Clínic-IDIBAPS, Barcelona, Spain; 6 Department of Hematology, Hospital Clinic -IDIBAPS, Barcelona, Spain; 7 Department of Autoimmune Diseases, Hospital Clinic, University of Barcelona, IDIBAPS, Barcelona, Spain; 8 University of Barcelona, Medical School, Barcelona, Spain; 9 Present Address: Clinical Pharmacology and Safety Sciences, BioPharmaceuticals R&D, AstraZeneca, Cambridge, UK; 10 Present Address: Garvan Institute of Medical Research, Sydney, Australia; 11 Present Address: Department of Gene Regulation, Stem Cells and Cancer Center for Genomic Regulation (CRG-PRBB), Barcelona, Spain

**Keywords:** Cancer microenvironment, B-cell lymphoma

## Abstract

Microenvironment contributes to follicular lymphoma (FL) pathogenesis and impacts survival with macrophages playing a controversial role. In the present study, using FL primary samples and HK follicular dendritic cells (FDC) to mimic the germinal center, together with mouse models, we have analyzed the three-way crosstalk of FL-FDC-macrophages and derived therapeutic opportunities. Ex vivo primary FL-FDC co-cultures (*n* = 19) and in vivo mouse co-xenografts demonstrated that FL-FDC crosstalk favors tumor growth and, via the secretion of CCL2 and CSF-1, promotes monocyte recruitment, differentiation, and polarization towards an M2-like protumoral phenotype. Moreover, FL-M2 co-cultures displayed enhanced angiogenesis, dissemination, and immunosuppression. Analysis of the CSF-1/CSF-1R pathway uncovered that CSF-1 was significantly higher in serum from grade 3A FL patients, and that high CSF-1R expression in FL biopsies correlated with grade 3A, reduced overall survival and risk of transformation. Furthermore, CSF-1R inhibition with pexidartinib (PLX3397) preferentially affected M2-macrophage viability and polarization program disrupting FL-M2 positive crosstalk. In vivo CSF1-R inhibition caused M2 reduction and repolarization towards M1 macrophages and antitumor effect cooperating with anti-CD20 rituximab. In summary, these results support the role of macrophages in FL pathogenesis and indicate that CSF-1R may be a relevant prognostic factor and a novel therapeutic target cooperating with anti-CD20 immunotherapy.

## Introduction

Follicular lymphoma (FL) is the most frequent indolent B-cell lymphoma, characterized by a variable clinical course. Several treatments lead to remissions, but the disease will eventually return, thus FL is still considered incurable [1]. FL arises from germinal center (GC) B cells that retain most of the phenotypic and functional features of the normal counterparts and a follicular organization reminiscent of GC supported by follicular dendritic cells (FDC) and T follicular helper (TFH) cells [2].

Next generation sequencing studies have deciphered the FL mutational landscape, highlighting the prominent relevance of genetic alterations affecting histone-modifying enzymes present in almost every FL patient [3]. In addition to these intrinsic abnormalities, the crosstalk between neoplastic B cells and non-neoplastic immune and stromal cells in the microenvironment plays an important role in disease severity, transformation, clinical outcome, and response to therapy [4, 5]. The first seminal paper by Dave et al. [6] found that immune signatures showed prognostic value. Subsequent immunohistochemical studies tried to transfer these findings into the clinical laboratory by associating the cellular composition of the microenvironment and its spatial distribution with the progression of the disease [7]. In this regard, the contribution of monocytes and macrophages to FL pathogenesis and the possible identification of suitable biomarkers with the potential to stratify FL patients remains a matter of debate [8–10]. This is likely due to the inherent complexity and plasticity of these cells subjected to microenvironmental cues that dictate their phenotype and function [11]. Despite these controversies in FL, it is remarkable that in other hematologic neoplasia such as Hodgkin lymphoma [12], and many solid tumors [13], macrophages have been linked to disease onset and/or progression. This observation sparked an interest to therapeutically target these plastic innate immune cells [14].

Blockade of colony-stimulating factor-1 (CSF-1) or its receptor CSF-1R represents a selective approach to manipulate macrophages in cancer patients [15] and some studies have explored this new therapeutic axis in some hematologic malignancies [16–19].

Here, we have analyzed the dynamic interaction between FL and its myeloid microenvironment. Using primary FL-FDC co-cultures to mimic the GC ex vivo and in vivo, we have demonstrated that FL-FDC niche orchestrates the recruitment and differentiation of monocyte to macrophages and their polarization into M2-like tumor associated macrophages (TAMs), which support FL survival, angiogenesis, and dissemination. In addition, we have found a dual role for CSF-1R as a prognostic marker and therapeutic target with the potential to increase the therapeutic benefit of standard immunotherapy.

## Methods

### FL microenvironment models

FL-FDC co-cultures were established with HK cells, a non-immortalized FDC cell line generated from normal tonsils which was kindly provided by Dr. Yong Sung Choi [20] at a 1:20 ratio (HK:FL), as previously described [21]. This cell line has been widely used to mimic lymphoma - LN stroma interaction [22–24].

FL-macrophage co-cultures were established at a 1:4 ratio (Mϕ:FL). Macrophages were derived from peripheral blood mononuclear cells (PBMCs) of healthy donors (Banc de Sang i Teixits, Barcelona). Blood samples were enriched in the monocyte population using RosetteSep (Human monocyte enrichment cocktail) (STEMCELL Technologies, Grenoble, France) and then cultured for 7 days with 100 ng/mL CSF-1 (Thermo Fisher Scientific, Waltham, MA, USA). When specified, differentiated macrophages were polarized to M1 (20 ng/mL IFNγ (Gibco, Thermo Fisher Scientific) + 100 ng/mL LPS (Sigma-Aldrich, St. Louis, MO)) or M2 (20 ng/mL IL4 (PeproTech, Rocky Hill, NJ)) for 24 h.

### Monocyte–macrophage differentiation and polarization analysis

Monocyte differentiation induced by FL was assessed co-culturing FL cell lines or primary cells (5 × 10^6^ cells/well) with monocytes from healthy donors at a 1:4 ratio (Mϕ:FL) for 7 days in standard six-well plates. The percentage of attached macrophages (CD11b + cells) was analyzed by flow cytometry and related to the positive control of differentiation (monocytes + CSF-1). LIVE/DEAD™ Fixable Aqua (Thermo Fisher Scientific) was used as a viability probe. To evaluate macrophage polarization, the expression of CD86 (M1-marker) and CD163 (M2-marker) was then analyzed in attached live macrophages (CD11b+, Aqua-) by flow cytometry as described in supplemental methods. In another set of experiments, monocyte differentiation was analyzed by imaging and co-culturing FL primary cells with monocytes labeled with CellTrace™ Far Red (Thermo Fisher Scientific). The number of attached macrophages (FarRed+) was analyzed by cell counting using Cytation™ 1 Cell Imaging Multi-Mode Reader (BioTek).

Detailed description of additional methods is included in supplementary material. These materials include, in vivo studies, gene expression profiling and bioinformatic analysis, flow cytometry, cytokine secretion assessment, HUVEC tube formation, adhesion to ECM, invasion, and migration assays, immunohistochemistry performance /quantification, and statistical analysis.

## Results

### FL-FDC crosstalk induces monocyte recruitment and their differentiation towards M2-like macrophages, favoring FL tumor growth

**Fig. 1:**
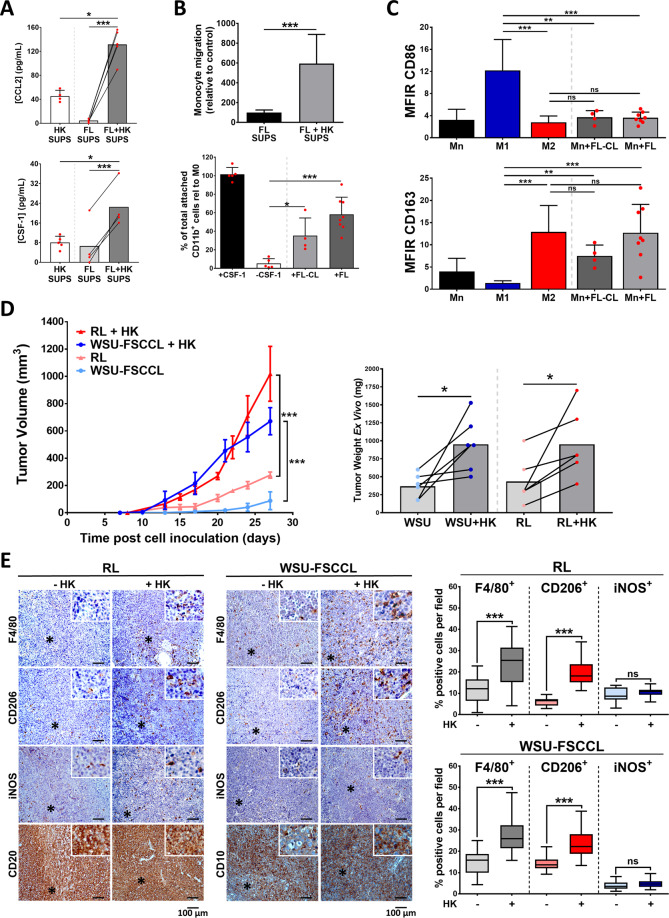
Follicular lymphoma induces monocyte recruitment, differentiation, and polarization to M2-like phenotype, increasing in vivo tumorigenicity. **A** CCL2 and CSF-1 secretion evaluated by ELISA in culture supernatants (SUPS) from primary FL or HK cells in monoculture and FL + HK co-cultures (48 h, *n* = 5). **B** Monocyte migration (*n* = 5) towards culture supernatants referred to FL monoculture supernatant (upper panel). Monocyte differentiation induced by FL was assessed co-culturing FL cell lines (FL-CL; *n* = 4) or primary cells (FL; *n* = 8) with monocytes for 7 days. Number of attached macrophages was analyzed by flow cytometry and related to the positive control of differentiation (Monocytes + CSF-1; lower panel). **C** CD86 (M1-marker) and CD163 (M2-marker) expression of attached macrophages at day 7 was analyzed by flow cytometry. **D** FL-HK mouse model was generated by subcutaneous inoculation of the FL cell lines WSU-FSCCL or RL with/without the FDC cell line HK in SCID mice (*n* = 8 mice per group). Graphs show tumor growth curves over time (left) and weights at the endpoint where mean ± SEM are plotted. **E** FFPE sections from representative FL and FL + HK tumors at the endpoint were stained with the pan-macrophage marker F4/80, the M2-marker CD206, and the M1-marker iNOS (magnification ×200). Representative images are shown (left panel). Quantification was done in the scanned slides using ImageJ software (right panel). Statistical significance was assessed using paired *t* test, Mann–Whitney test and the multiple *t*-Tests (Holm–Sidak method) for differences in tumor growth over time. Bars represent the SD except for **D**, representing SEM.

We first acknowledged that supernatants of FL-FDC co-cultures were enriched in the monocyte chemo attractant CCL2, and in the pivotal cytokine for monocyte activation and differentiation CSF-1, compared to the supernatants of FL or HK cells in monoculture (Fig. 1A). Accordingly, FL-HK co-culture supernatants recruited a significantly higher number of monocytes than monoculture supernatants (Fig. 1B, upper panel). Moreover, HK cells support primary FL patient cell viability in agreement with our previous results in FL [21] and other lymphomas [22–24] (Fig. S1A). Similar results were found in FL-monocytes co-cultures, supporting the notion of a three-way positive crosstalk (Fig. S1B).

We next sought to determine whether FL might promote monocyte–macrophage differentiation. Monocytes from healthy donors were then put in direct contact with FL cell lines (FL-CL) and primary cells from FL patients (FL). Of note, a significant number of monocytes differentiated into macrophages in these co-cultures, and more prominently when primary FL cells were used (Fig. 1B lower panel and Fig. S1C). Using M1 and M2-polarized macrophages as internal controls, we determined that FL polarizes macrophages towards M2-like macrophages, as they showed higher CD163 than CD86 surface expression (Fig. 1C).

We next validated in vivo these in vitro results using an FL-HK co-xenograft generated with two different FL cell lines (RL and WSU-FSCCL). The histological evaluation of the tumors showed a subcutaneous mass comprised of medium and large lymphocytes, which a diffuse distribution pattern with some areas vaguely nodular and a variable presence of cells with macrophage and (follicular) dendritic shape. In some areas the tissue had undergone necrosis but most of the areas were viable. The lymphocytes were of medium and large size, with slightly irregular nuclear membrane and small nucleoli, and homogeneously positive for B-cell markers such as CD20 (RL) or the germinal center marker of CD10 (WSU-FSCCL). When comparing tumors with or without FDC cells, we observed that FDC HK cells support lymphomagenesis as indicated by the increase in the tumor size of FL-HK tumors compared to FL cell lines alone (Figs. 1D and S1D), in agreement with previous results in other GC lymphomas [22]. Likewise, the percentage of positive cells for the proliferation marker pH3 (Fig. S1E) was higher in FL-HK tumors.

It is noteworthy that the FL-HK tumors were significantly more infiltrated with mouse macrophages than FL tumors, identified with the pan-macrophage marker F4/80 (Fig. 1E). In these tumors, the macrophages not only increased in number but also in shape acquiring a dendritic form-like shape. Supporting our in vitro results, the vast majority of these infiltrated macrophages expressed the mouse M2-marker CD206, while a minor proportion expressed the mouse M1-marker iNOS (Fig. 1E), both markers commonly used in mouse models [25].

In summary, FL-HK niche favors tumor growth and monocyte/macrophage recruitment and their polarization towards a clear M2-like protumoral phenotype.

### M2 macrophages shape FL cancer hallmarks

We next explored if macrophage infiltration was a hallmark of FL, and we compared by CIBERSORT [26] (detailed information in supplemental methods) the presence of several immune populations in normal tonsils (NTS) and follicular lymphoma lymph nodes (FL-LN), using publicly available data. FL-LNs were enriched in T cells and M1 and M2 macrophages compared to NTS and more prominently M2 than M1 (Fig. S2A). To characterize how M2-contribute to FL tumorigenesis, we established ex vivo co-cultures of primary FL patients’ cells from LN biopsies with M2 macrophages generated from monocytes of healthy donors. FL-M2 co-cultures significantly (*p* = 0.0002) increased the viability of FL cells (Fig. S2B). GEP of purified B cells from FL-M2 co-cultures indicated that M2 significantly modified the FL transcriptome. A total of 177 mRNAs were upregulated in FL cells co-cultured with M2 macrophages (fold change (FC) > 2 and *p* < 0.05). These genes belong to fundamental biological cancer pathways as cell proliferation, movement, adhesion, cytokine signaling, and immune response (Fig. S2C). Gene set enrichment analysis (GSEA) of the whole expression data set of FL-M2 co-cultures vs FL monocultures uncovered a striking enrichment of genes related to angiogenesis, integrin pathways, leukocyte migration, and cytokine-receptor interaction among others (Table S2). Enrichment plots and heatmaps of the corresponding leading edges of representative upregulated pathways that illustrate these gene expression changes in FL cells produced by M2 macrophages are shown in Figs. S2D and 2. A complete list of enriched gene sets is provided in Table S3.

**Fig. 2:**
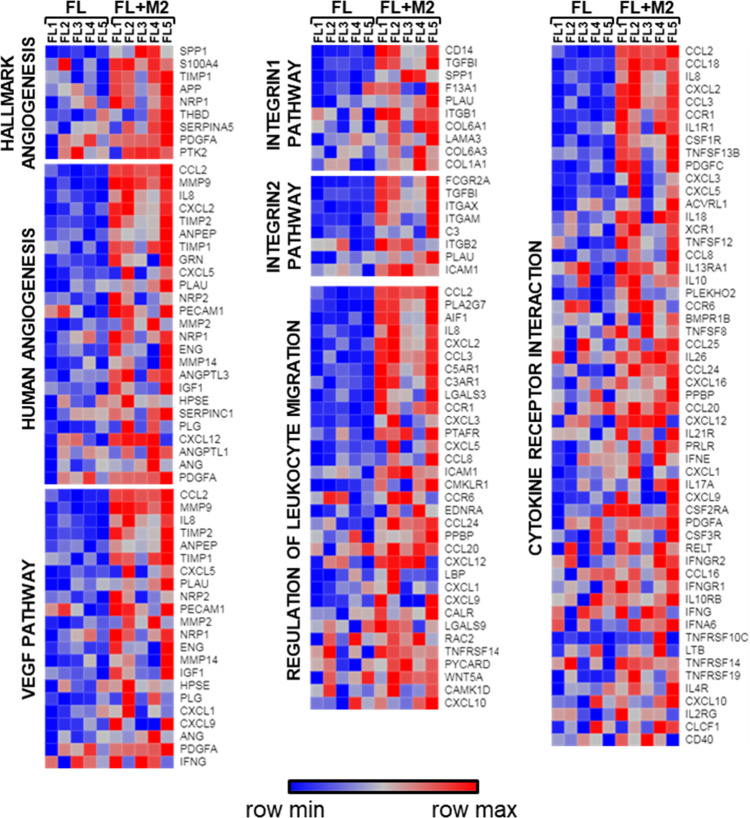
M2 macrophages induce up-regulation of gene sets related to migration, invasion, and angiogenesis in FL patient samples. Primary FL cells (*n* = 5) were co-cultured (48 h) with M2 macrophages at 4:1 ratio, generated from peripheral blood monocytes of healthy donors (100 ng/mL CSF-1, 1 week). Purified B cells were then subjected to GEP. Data mining analysis was done with GSEA software (FDR < 0.05, NES > 1.5). Representative heatmaps of adhesion, invasion, angiogenesis, and cytokine secretion gene sets are shown. The leading edge of each gene set is displayed in a heat map.

Next, we validated these results at a functional level, focusing on two relevant processes in lymphoma, such as angiogenesis and cell dissemination, that includes the steps of adhesion and invasion.

**Fig. 3:**
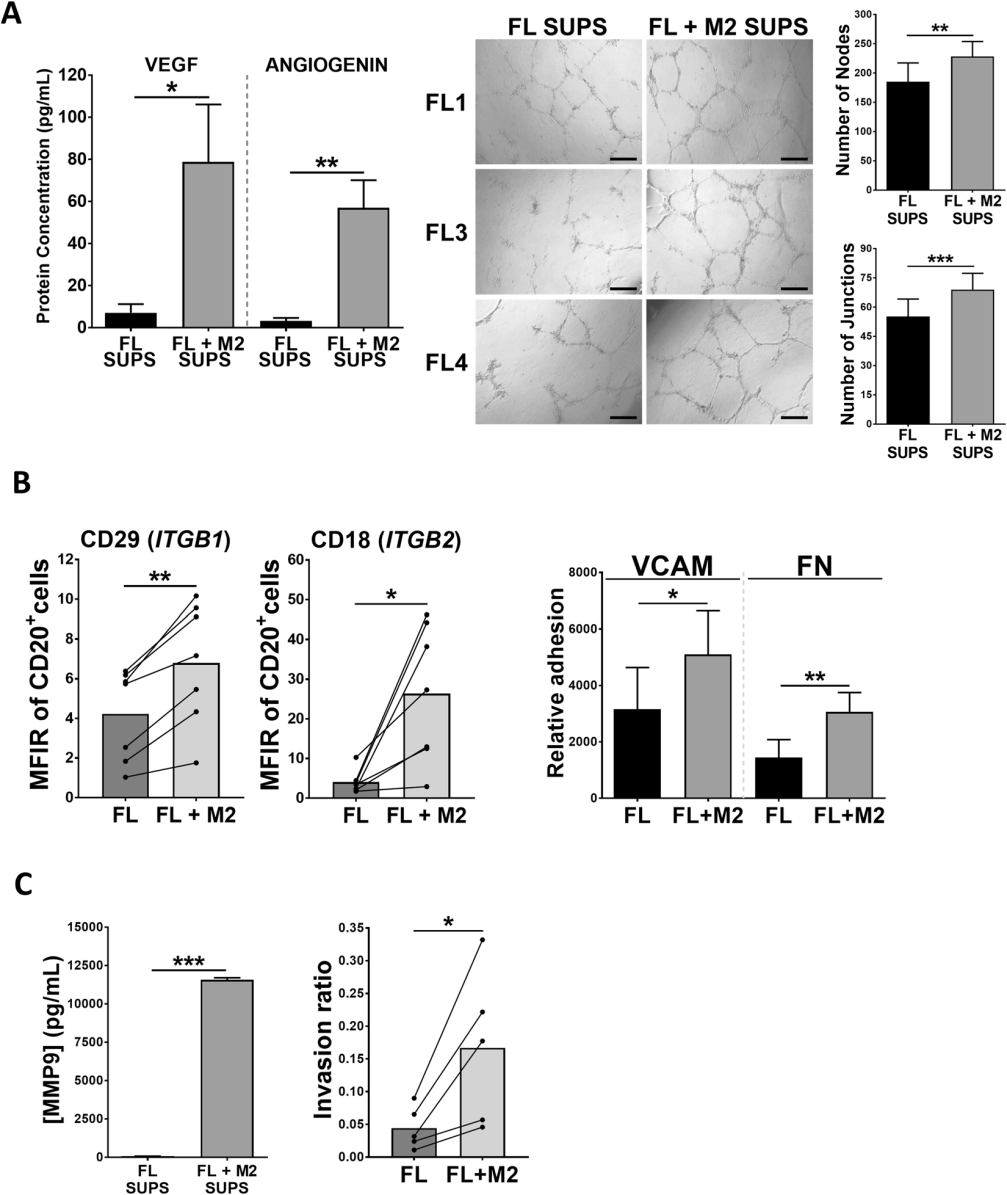
M2 macrophages increases FL cells angiogenesis, adhesion, invasion, and related cytokine secretion. **A** VEGF-A and angiogenin were quantified in supernatants (SUPS) from FL monocultures and FL-M2 co-cultures (48 h; left panels). Those supernatants were also used in a HUVEC tube formation assay (right panels). The number of nodes and junctions per field were obtained using ImageJ software with Angiogenesis Analyzer plugin. The data is representative of 3 FL patients. **B** Surface expression of CD29 (Integrin beta-1, ITGB1) and CD18 (Integrin beta-2, ITGB2) was analyzed by flow cytometry in FL primary samples (*n* = 7) after 48 h of culture with or without M2 (left panels). FL cells from FL monocultures or FL-M2 co-cultures (48 h) were subjected to an adhesion assay to VCAM or Fibronectin (FN) (n = 5) (right panel). **C** MMP9 vas quantified in supernatants (SUPS) from FL monocultures or FL-M2 co-cultures (*n* = 5) and the corresponding FL cells were recovered and subjected to an invasion assay for 24 h.

We first assessed the presence of proangiogenic factors in the supernatants of FL-M2 co-cultures and found a significant enrichment of VEGF-A and angiogenin. These supernatants were then used in a tube formation assay with HUVEC and showed that those from FL-M2 co-cultures significantly increased the number of nodes and junctions compared to those from FL monocultures (Fig. 3A).

Furthermore, M2 macrophages increased the expression of a set of integrins (Fig. 2) that was validated at protein level by flow cytometry for ITGB1 (CD29) and ITGB2 (CD18) (Fig. 3B). Of note, CD18 has been related to advanced stages in NHL [27]. ITGB1 (CD29) together with ITGA4 (CD49d) integrates the lymphocyte homing receptor VLA-4 with affinity for VCAM and Fibronectin (FN). In accordance, those FL cells from FL-M2 co-cultures exhibited superior adhesion capacities to these extracellular matrix components (Fig. 3B).

Adhesion represents a precedent step for cell migration/invasion onset [28]. GSEA analysis revealed that M2 macrophages induced an enrichment in FL cells of gene sets related to the dissemination process, including several related to migration (ie, chemokines and chemokine receptors, chemokine signaling, and leukocyte chemotaxis) or invasion (epithelial mesenchymal transition; Tables S2 and S3). We validated by ELISA the increased secretion of CXCL12/SDF-1 and MMP9 (Figs. 3C and S3), mirroring GEP data. Likewise, an invasion assay using Matrigel coated inserts, proved that those FL cells that had been in contact with M2 macrophages exhibited higher invasion capacities (Fig. 3C).

In addition, our results indicate that M2 macrophages induced an enrichment in FL cells of cytokines and chemokines that may attract other immunes populations, reshaping the lymphoma niche, such as CCL2 and CCL3 and IL-8 (Fig. S3), which may facilitate the recruitment of monocytes and neutrophils [29–31]. Moreover, the levels of immunosuppressive cytokines IL-10 and IL-8 were prominent in these co-cultures, together with the chemokine CCL18, with demonstrated pro-metastatic properties [32] (Fig. S3).

Altogether, these results illustrate the power of infiltrating M2 macrophages to increase angiogenesis, dissemination, and immunosuppression in the FL niche.

### Macrophage depletion hampers the outgrowth of FL-HK tumors and reduces FL dissemination

**Fig. 4:**
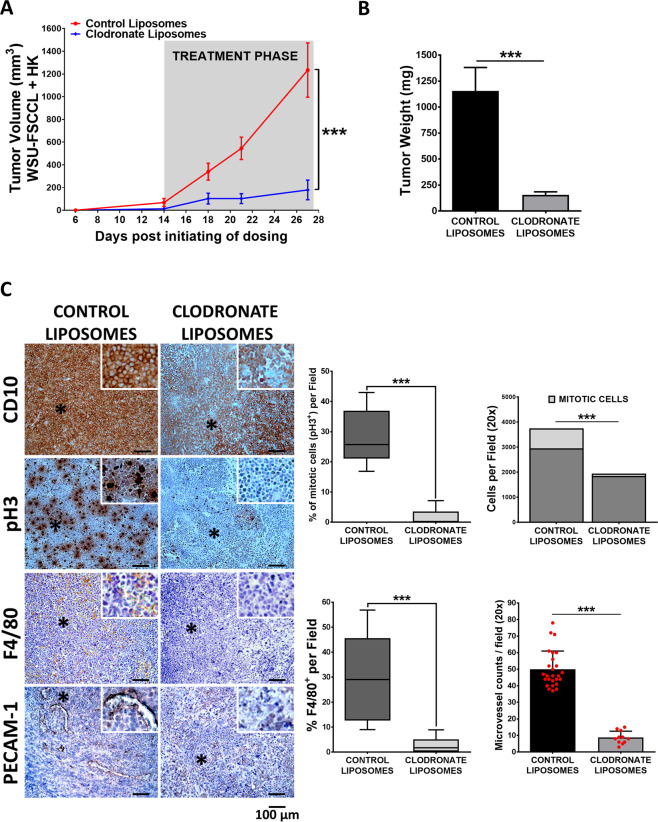
Macrophage depletion hampers the outgrowth of FL-HK tumors. **A** WSU-FSCCL + HK co-xenografts were randomized by weight into two groups (*n* = 9–10 mice per group). At day 14 each group received an intratumoral injection of 100 mL/kg clodronate liposomes or an equal volume of control empty liposomes, 2 days per week, during 14 days. Graph shows tumor growth curves over time where mean ± SEM are plotted. Statistical significance was assessed by the multiple t-Tests (Holm–Sidak method). **B** Tumors were excised and weighed at the endpoint (day 27). **C** FFPE sections from tumors of both groups at the endpoint were probed for the GC-B-cell marker CD10, the proliferation marker pH3, the pan-macrophage marker F4/80 and the microvessel marker PECAM/CD31 (magnification ×200). Representative images are shown (left panel). Quantification was done in the scanned slides using ImageJ software (right panel). Statistical differences between groups were assessed by Mann–Whitney test. Bars represent the SD.

In order to demonstrate the implication of macrophages in FL pathogenesis and its possible relevance s targets for FL therapy, we chose to deplete them in vivo using liposomal clodronate, a commonly used approach [33], and evaluate its impact on tumor growth and dissemination. Using the same model as in Fig. 1 (FL-HK co-xenograft), we demonstrated that macrophage depletion implies an almost complete inhibition of tumor growth (Figs. 4A, B and S4A). IHC analysis of tumor sections (Fig. 4C) confirmed complete macrophage depletion (F4/80+), and a significant reduction of mitotic index indicated by a complete reduction of pH3^+^ cells, a hallmark of active cell proliferation [34]. In addition, clodronate-treated tumors appeared scarcely vascularized (Fig. S4A) with low PECAM-1 staining (Fig. 4C), supporting the role of macrophages in the angiogenic process.

To assess the effect of macrophages on FL dissemination, we used a systemic mouse model where FL cells (WSU-FSCCL-Luc) were intravenously inoculated (Fig. S4B). At the endpoint, the control liposome group showed systemic disease, with spleen, brain, and BM infiltration in agreement with previous results [35]. Mice treated with clodronate liposomes showed a significant reduction of tumor load and dissemination to BM and brain as quantified by bioluminescence (Fig. S4B; *p* = 0.002 control vs clodronate (day 26)). Similarly, spleen size decreased in clodronate-treated mice (Fig. S4C, *p* < 0.001) mostly caused by a reduced FL cell infiltration (CD10+) together with macrophage depletion (F4/80+; Fig. S4C).

Thus, macrophages contribute to FL tumor growth and dissemination, suggesting that these immune cells may constitute new targets to modulate FL microenvironment support.

### CSF-1R expression in FL is associated with reduced overall survival and histological transformation

As we have shown above, CSF-1 increased in FL-FDC co-cultures (Fig. 1B) and may contribute to monocyte recruitment and differentiation in the FL-LN microenvironment. As CSF-1/CSF-1R pathway is fundamental monocyte/macrophage differentiation and activation [36], we next study its possible relevance in FL pathogenesis and derived therapeutic opportunities.

**Fig. 5:**
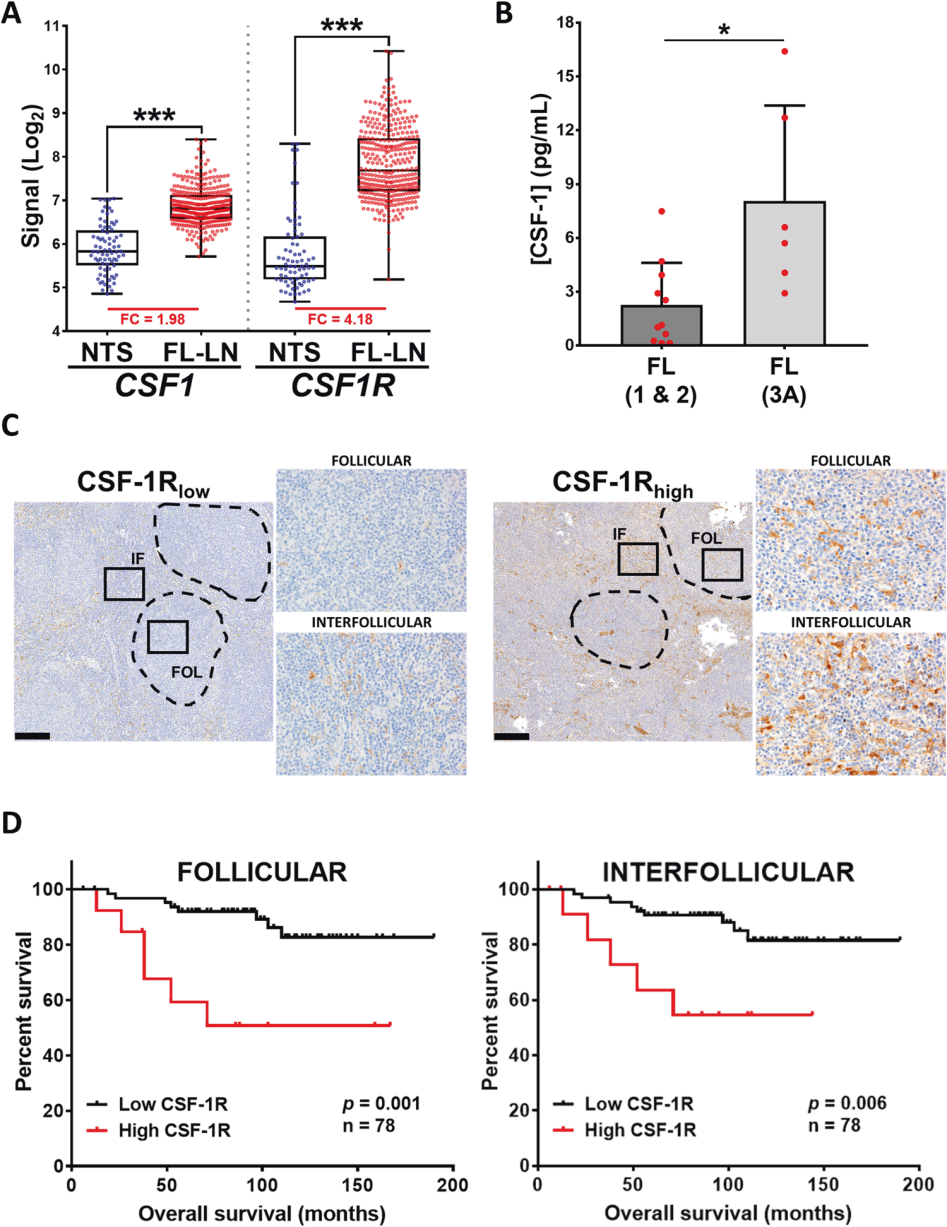
CSF-1/CSF1-R expression correlates with grade and impacts overall survival of FL patients. **A** Expression of *CSF1* and *CSF1R* in FL-LN (*n* = 362) compared to normal tonsils (NTS; *n* = 75) according to GEP public databases. Mann–Whitney test. **B** Concentration of CSF-1 protein evaluated by ELISA in serum of grade 1 & 2 (*n* = 11) and grade 3A (*n* = 6) FL patients. Mann–Whitney test. **C** IHC analysis of CSF-1R expression was performed in FFPE biopsies of FL patients (*n* = 78; grade 1–2 (*n* = 56) grade 3A (*n* = 22). Representative images of FL cases with high and low CSF-1R expression are shown at low (×40) and high (×200) magnification of both follicular (FOL) and interfollicular (IFOL) areas. **D** Kaplan–Meier plot of overall survival of FL patients grouped on the basis of CSF-1R expression (*n* = 78). MaxStat software was used to determine the cut-off value producing the maximum score in log-rank test. Cut-off values were 8.335 for follicular compartment and 21.379 for interfollicular. *P*-value was obtained from the overall log-rank test.

We first queried publically available databases for the expression of CSF1-R and its ligand CSF-1 in FL-LN and normal tonsils (NTS). Both *CSF1* and *CSF1R* expression were overrepresented in FL-LN compared to NTS (Fig. 5A). The specificity and sensitivity of these genes to identify the FL-LN group was calculated by a ROC curve (Fig. S5A). Next, we quantified CSF-1 expression in serum from FL patients (*n* = 17, Table S1) by ELISA and CSF-1R by IHC in a series of FL patient samples from our institution (*n* = 78, Table 1). We found that CSF-1 expression was significantly enriched in serum from grade 3A FL patients (Fig. 5B). Likewise, CSF-1R expression was variable among FL patient biopsies and was detected both in the follicular and interfollicular areas being more intense in the latter (Fig. 5C). In agreement with CSF-1 results, the expression of CSF-1R was higher in grade 3A compared to grade 1 and 2 FL cases, both in follicular and interfollicular areas (Fig. S5B and Table 1). Finally, we explored the association of CSF-1R with clinical variables. Interestingly, we found a correlation of high CSF-1R expression with reduced OS and risk of transformation, both in follicular (cut-off = 8.335) and interfollicular (cut-off = 21.379) areas (Fig. 5D and Table 1). A correlation of CSF-1R with PFS (Fig. S5C), ECOG performance status and spleen involvement was found just for follicular CSF-1R expression (Table 1).

**Table 1:** Clinical features of FL patients tested for CSF-1R expression and association of CSF-1R with clinical variables.

Characteristics	All patients *n* = 78	CSF1R					
		Follicular expression			Interfollicular expression		
		Low	High	*P* value	Low	High	*P* value
		*n* = 65 (%)	*n* = 13 (%)		*n* = 65 (%)	*n* = 13 (%)	
Age > 60 years, *n* (%)	25	20 (31)	5 (38)	NS	22 (34)	3 (23%)	NS
Sex (M/F)	32/46	27/38	5/8	NS	26/39	6/7	NS
Histological grade, *n* (%)							
Grade 1–2	56	50 (77)	6 (46)	–	50 (77)	6 (46)	–
Grade 3A	22	15 (23)	7 (54)	**0.04**	15 (23)	7 (54)	**0.04**
B symptoms, *n* (%)	13	10 (15)	3 (23)	NS	9 (14)	4 (31)	NS
ECOG ≥ 2, *n* (%)	6	3 (5)	3 (23)	**0.05**	4 (6)	2 (15)	NS
Bulky disease, *n* (%)	24	20 (31)	4 (31)	NS	17 (26)	7 (54)	NS
Stage III-IV disease, *n* (%)	48	38 (58)	10 (77)	NS	38 (54)	10 (77)	NS
Spleen involvement, *n* (%)	17	11 (17)	6 (46)	**0.03**	14 (22)	3 (23)	NS
Hemoglobin <12 gr/L, *n* (%)	14/75	10 (15)	4 (31)	NS	11 (17)	3 (23)	NS
High serum LDH, *n* (%)	20/74	17 (26)	3 (23)	NS	16 (25)	4 (31)	NS
High serum B2m, *n* (%)	38/73	29 (45)	9 (69)	NS	31 (48)	7 (54)	NS
High risk FLIPI, *n* (%)	21/75	15 (23)	6 (46)	NS	16 (25)	5 (38)	NS
Histological transformation, *n* (%)	5	2 (3)	3 (23)	**0.0078**	2 (3)	3 (23)	**0.0025**

*ECOG* Eastern Cooperative Oncology Group, *FLIPI* Follicular Lymphoma International Prognostic Index. Statistically significant *p*-values are in bold.

Cut-off follicular expression CSF1R: 8.335.

Cut-off interfollicular expression CSF1R: 21.379.

In summary, we have identified a for the first time a correlation between CSF-1R macrophage expression with FL grade 3A, reduced OS and risk of transformation, justifying the possibility to modulate macrophage activity in FL, and highlighting CSF-1R pathway as a new target for therapy.

### CSF-1R blockade reprograms M2 to M1 macrophages and disrupts FL-M2 macrophages crosstalk

We first determined the expression of CSF-1R by flow cytometry in M1 and M2 macrophages generated from PBMC of healthy donors. CSF-1R was detected both intracellular and on the surface of M1 and M2 macrophages, although it was more prominent in M2 macrophages, which also expressed a higher proportion of CSF-1R in their plasma membrane (Fig. S6A). We next assessed the effect of the CSF-1R inhibitor PLX3397 (pexidartinib). This inhibitor blocks the tyrosine kinase activity of the receptor with significant selectivity over other kinases at the doses used (50 nM). PLX3397 is being tested in clinical trials for several types of cancer including a number of hematologic malignancies [37].

**Fig. 6:**
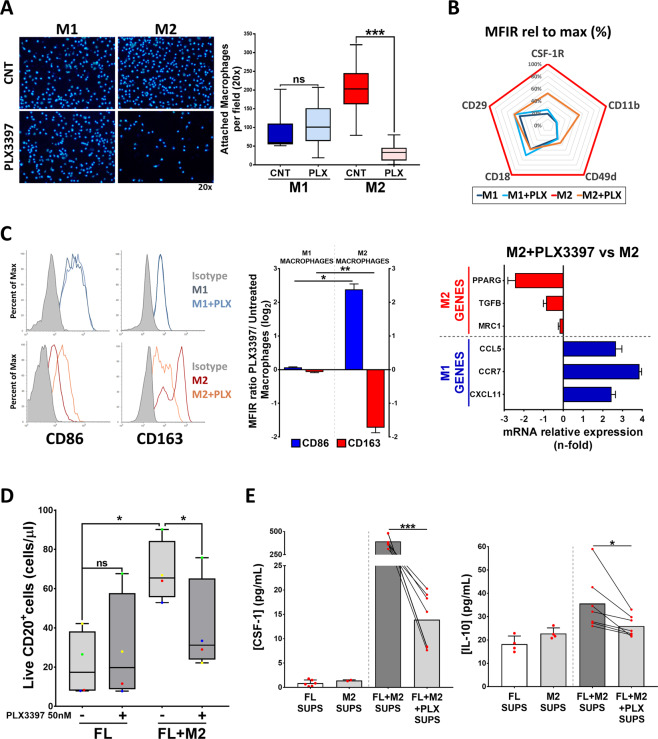
CSF-1R blockade with PLX3397 inhibits M2 macrophage adhesion and reprograms M2 macrophages to M1, disrupting crosstalk between FL cells and M2 macrophages. **A** M0 macrophages were detached and pre-treated for 1 h with 50 nM PLX3397, then seeded for 24 h in the presence of CSF-1 + IFNγ + LPS in the case of M1 macrophages or CSF-1 + IL4 for M2 macrophages. Attached macrophages were  stained with Hoechst 33342 and quantified by Image J. **B** CSF-1R and integrin expression profile of M1 and M2 macrophages after PLX3397 (50 nM, 48 h) treatment. **C** M1 and M2 selected markers were analyzed by flow cytometry (CD86 and CD163) and RT-qPCR (PPARG, TGFB, MRC1, CCL5, CCR7, and CXCL11) in M2 macrophages treated or not with PLX3397 (50 nM, 48 h). **D** Primary tumor cells from FL patients (*n* = 4) were treated with PLX3397 (50 nM, 48 h) in co-culture or not with M2 macrophages (CSF-1 + IL4). Total number of live (LIVE/DEAD Aqua^−^) CD20 + cells is shown. **E** Concentration of CSF-1 and IL-10 determined by ELISA in supernatants from 6F. Statistical significance was assessed using paired *t* test or Mann–Whitney test.

CSF-1R inhibition by PLX3397 totally impedes the differentiation of monocytes to macrophages (Fig. S6B). PLX3397 did not affect M1 macrophages but significantly reduced the viability of M2 macrophages (Fig. S6C) and likewise interfered with the adhesion process preferentially of M2 macrophages (Fig. 6A). In this regard we observed that M1 macrophages (dim CD11b, CD18, CD49d, and CD29) exhibited a different integrin profile than M2 macrophages (high CD11b, CD18, CD49, and CD29) and that PLX3397 reverted this profile towards an “M1-like” phenotype (Fig. 6B). Interestingly PLX3397 also decreased CSF-1R expression in M2 macrophages. These results led us to examine the effect of PLX3397 on the polarization state of both M1 and M2 macrophages, by means of the flow cytometry surface markers CD86 and CD163. CSF-1R inhibition significantly decreased CD163 expression and increased CD86 of M2 macrophages, while their expression remained unchanged in M1 macrophages (Fig. 6C). Likewise, the expression of M2-like genes (PPARG, TGFB, and MRC1) decreased and M1-like (CCL5, CCR7, and CXCL11) increased in PLX3397-treated M2 macrophages.

Of note, CSF-1R inhibition hampered FL-M2 positive crosstalk whereas it did not affect the viability of lymphoma cells in monoculture (Figs. 6D and S6E). Interestingly, supernatants from PLX3397-treated M2 macrophages showed inferior pro-survival power than untreated ones on WSU-FSCCL cells (Fig. S6D). The analysis of co-culture supernatants revealed that PLX3397 depleted CSF-1 and reduced IL-10 secretion (Fig. 6E) whereas IL-34 (the alternative CSF-1R ligand) remained unaffected (Fig. S6F).

In summary, CSF-1R inhibition preferentially affects M2-macrophage viability and polarization program and disrupts FL-M2 positive crosstalk.

### CSF-1R inhibition and rituximab cooperate in vivo by hampering tumor outgrowth, limiting macrophage infiltration, and promoting M2-M1 reprogramming

**Fig. 7:**
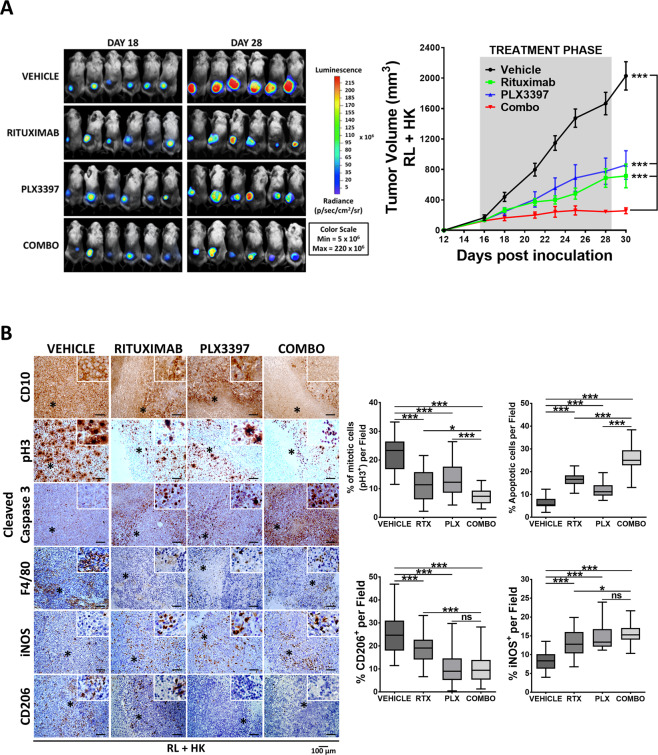
Rituximab and PLX3397 combined treatment hampers in vivo tumor outgrowth. RL (luciferase)-FDC co-xenografts were randomized when tumor volumes reached 200 mm^3^ (*n* = 6 mice per group) and treatments started (day 16). Mice were treated with PLX3397 or vehicle (50 mg/kg, po), in a 5-on 2-off schedule and/or Rituximab (20-10-10 mg/kg ip) once a week till the endpoint (day 31). **A** Bioluminescence images of the four treatment groups at days 18 and 28 (left). Graph shows tumor growth curves over time where mean ± SEM are plotted (right). Statistical significance was assessed by the multiple t-Tests (Holm–Sidak method). **B** FFPE sections from representative tumors of the four groups were stained with the GC marker CD10, the proliferation maker pH3 and the apoptosis marker active caspase-3 together with the pan-macrophage marker F4/80, the M2-marker CD206, and the M1-marker iNOS (magnification ×200). Representative images are shown (left panel). Quantification was done using ImageJ software (right panel). Statistical differences between groups were assessed by Mann–Whitney *t* test. Bars represent the SD.

We next sought to determine if anti-CSF-1R therapy could increase the efficacy of standard lymphoma immunotherapy (anti-CD20 rituximab). To achieve this aim, we set up an in vivo experiment using the RL cell line sensitive to rituximab [38] in the co-xenograft model of RL-HK described in Fig. 1D. Mice treated with the single agents showed a significant reduction of tumor size assessed by bioluminescence, tumor volume (*p* < 0.001 control vs PLX3397; *p* < 0.001 control vs rituximab at endpoint; Fig. 7A) and tumor weight (*p* < 0.01 control vs PLX3397; *p* < 0.01 control vs rituximab; Fig. S7A). The significant activity of PLX3397 in monotherapy agrees results obtained in depletion experiments with clodronate liposomes shown above (Figs. 4 and S4) and supported the prominent role of macrophages in FL. Furthermore, the combination of both agents completely stopped tumor growth when comparing the endpoint (day 30) with the day of treatment initiation (day 16), significantly increasing single agent activity (*p* < 0.05 rituximab vs rituximab+PLX3397; *p* < 0.001 PLX3397 vs rituximab+PLX3397; Figs. 7A and S7A). IHC analysis of the GC marker CD10 or CD20 to identify the RL cells confirms the depletion of tumor cells (Figs. 7B and S7C). Analysis of the proliferation marker pH3 of tumor sections by IHC confirmed the cooperation between both agents (Fig. 7B). Moreover, analysis of active caspase-3 indicated that both agents engage significant apoptosis that was further increased by the combination. Macrophages decreased with all treatments (F4/80+, Fig. 7B) and more importantly, the combination favored the M1-polarization of remaining infiltrating macrophages, as indicated by an increase in the M1-marker iNOS and a decrease in the M2-marker CD206 (Figs. 7B and S7B).

In summary, targeting the CSF-1R axis cooperates with rituximab in terms of inhibition of tumor growth, B-cell depletion, and M2 macrophage infiltration.

## Discussion

FL arises from developmentally blocked GC-B cells that retain phenotypic and functional features of normal counterparts and a follicular organization reminiscent of GC [2]. Microarray analysis of FL tumor biopsies at diagnosis demonstrated that the clinical behavior of FL might be associated with the GEP of non-malignant cells in the tumors rather than from malignant B cells. Thus, an immune response enriched in genes expressed in macrophages and/or FDC was related with worse outcome [6]. Based on these results, we were interested in characterizing the three-way crosstalk of FL-FDC-macrophages with the aim of disrupting this protumoral crosstalk for therapeutic purposes. We have established primary FL-FDC co-cultures using FL samples mostly from LN biopsies (composed mainly by tumor B cells and several T subpopulations including TFH, regulatory, and cytotoxic T cells) and supportive FDC (HK cell line) generated from tonsils of normal donors previously used to mimic de GC [22] and promote B-cell survival [23]. Using this primary co-culture model, we previously reported that FL-FDC crosstalk shapes key lymphoma features including adhesion, dissemination, angiogenesis, and ECM remodeling, among others [21, 39]. In the present study, we have discovered that FL-FDC crosstalk favors tumor growth onset, and through the secretion of CCL2, as previously reported in FL BM microenvironment [40], and CSF-1 induces the recruitment of monocytes and their differentiation-polarization towards a clear M2-like protumoral phenotype, both in vitro (CD163^high^ CD86^low^) and in vivo (CD206^high^ iNOS^low^), cooperating in providing long-term protumoral support. Moreover, and despite the known limitations of FL cell lines, we have been able to validate these in vitro findings with primary FL cells, in mouse co-xenografts of FL cell lines and HK cells.

Macrophages participate in tumor initiation as a part of anti-inflammatory response and are immune activated in this scenario [41]. However, once tumors are established, those macrophages are educated to become protumoral, expressing characteristic surface molecules, such as the hemoglobin scavenger receptor CD163 and macrophage mannose receptor 1 (also known as CD206) [42] as we have confirmed in our in vitro and in vivo systems (Fig. 1). These TAMs demonstrate properties related to stimulation of angiogenesis, suppression of adaptive immunity, and promotion of cancer growth and metastasis [43]. Using co-cultures of primary FL cells and in vitro generated M2 macrophages, we have been able to recapitulate these paradigmatic hallmarks in FL [44], validated by transcriptomic and functional studies. Co-culture supernatants were dramatically enriched in proangiogenic factors (VEGF and angiogenin), chemo attractants of monocytes and neutrophils (CCL2, CLL3 and IL-8), and factors promoting cell dissemination to distant sites (CXCL12, CCL18, and MMP9). These results prompted us to explore the effect of macrophage depletion and reprogramming in FL pathogenesis. Depletion experiments with liposomal clodronate impaired tumor growth (>90% of reduction in tumor size) in heterotypic models with subcutaneous FL-HK tumor, and hampered dissemination in a systemic FL model. Similar results were shown in studies done in CLL transplantation systems in immune-suppressed mice or using the TCL-1 transgenic mice [17, 45]. Thus, our results represent the first proof of concept to consider macrophages as targets for therapy in FL.

Nevertheless, the prominent results obtained with clodronate, should be interpreted with caution, as macrophage depletion may not be the best approach. Macrophages have dual roles in cancer depending on the specific scenario, and show antitumor functions in some therapeutic settings, such as in antibody therapy via ADCC and ADCP [46] or in drug-induced immunogenic cell death [47]. Consistent with these observations, although high TAM density has been associated with an unfavorable prognosis in FL [48], high TAM infiltration was associated with a favorable outcome in patients treated with rituximab-containing regimens [9]. Hence, for therapeutic purposes in a tumor scenario, macrophages must be retuned, not totally depleted as happens with clodronate liposomes (Figs. 4 and S4). With this idea in mind, we explored the CSF-1/CSF-1R pathway in FL. CSF-1 is a monocyte attractant as well as a macrophage survival and polarization factor that drives TAM differentiation towards an immunosuppressive, tumor promoting ‘M2-like’ phenotype. CSF-1R is exclusively expressed by cells of the monocytic lineage and, therefore, the CSF-1/CSF-1R axis has been extensively investigated in tumor models and is paradigmatic of the TAM-cancer cell interaction [49, 50].

We first acknowledged that the mRNA expression of both the ligand and the receptor were enriched in FL-LN compared to normal tonsils. Additionally, we found increased levels of CSF-1 in the serum of grade 3A FL patients. Although the number of patient serum available was low (*n* = 17), these results are meaningful because they are concordant with those reported in CLL, where *CSF1* transcripts are significantly more abundant in progressive CLL patients [51]. Likewise, overexpression of CSF-1 is associated with poor prognosis in breast, ovarian, endometrial, prostate, and hepatocellular [52–54]. Importantly, the frequency of both follicular and interfollicular CSF-1R^+^ TAMs correlate with reduced OS, similar to results found in HL using the same CSF-1R antibody [55], grade 3A and risk of transformation, in FL patients homogenously treated with R-CHOP at our institution. These novel results, if confirmed in independent FL patient cohorts, may have implications in upfront treatment decisions and patient stratification.

Regarding CSF-1R/CSF-1 axis as a target for therapy, we have demonstrated that CSF-1R inhibition by PLX3397 affected more M2 than M1 macrophages, and induces their repolarization towards an M1-like phenotype, in accord with published results in glioma [56, 57]. More importantly, CSF-1R inhibition disrupted M2-derived pro-survival crosstalk in FL cells and reduced the immunosuppressive factors CSF-1 and IL-10, conforming a less permissive microenvironment. Interestingly, these results were perfectly in vivo transferable, where CSF-1R inhibition alone was sufficient to cause a significant reduction (40%) of tumor growth. This is interesting as in other tumor models, such as breast cancer [58], the sole inhibition of CSF-1R does not translate into antitumor activity but cooperates with cytotoxic agents. In our model CSF-1R inhibition also cooperates with standard antibody therapy anti-CD20, hampering tumor growth. As depicted in Fig. 7, the volume of tumors treated with the combination remained constant (~200 mm^3^) from the initiation of treatment onwards, arguing for a stabilization of the disease.

In conclusion, our results support the role of M2 macrophages in FL pathogenesis and suggest that therapies manipulating FL-M2 crosstalk may be a new strategy for those patients with high macrophage infiltration, especially in combination with anti-B-cell therapies.

## Supplementary information

Valero JG.Supplemental material
