# Supplementary material for: The receptor of the colony-stimulating factor-1 (CSF-1R) is a novel prognostic factor and therapeutic target in follicular lymphoma
Source: Leukemia. 2021 Mar 17;35(9):2635–49. doi: 10.1038/s41375-021-01201-9 (PMC8410584; doi:10.1038/s41375-021-01201-9)
Supplement: Supplementary file 1 — Valero JG.Supplemental material [file 41375_2021_1201_MOESM1_ESM.pdf]

## SUPPLEMENTAL METHODS

### FL cell lines and primary cells

FL cells were isolated from lymph nodes, peripheral blood, tonsil or paravertebral mass from a cohort of 19 patients, and serum from 17 patients diagnosed according to the World Health Organization (WHO) classification criteria at the Hospital Clinic of Barcelona (Table S1). The Ethics Committee of Hospital Clínic approved this study (IRB# HCB/2018/0397) and patients signed written informed consent.

FL cell lines DOHH-2, RL, SC-1 and WSU-FSCCL were obtained from DSMZ (Braunschweig, Germany). RL and WSU-FSCCL cell lines expressing luciferase (RL-luc, WSU-FSCCL-luc) were generated *via* retroviral transduction. Retroviral vectors containing full-length pLHCX-luc were kindly provided by Dr. Bofill-De Ros (IDIBAPS, Barcelona, Spain) (see below method description).

Cell lines and patient derived cells were cultured in RPMI-1640 supplemented with 10% FBS, 2mmol/L L-glutamine, 50 µg/mL penicillin/streptomycin (Thermo Fisher Scientific) and were maintained in a humidified atmosphere at 37ºC containing 5% $CO_2$. Normocin (100 µg/mL; InvivoGen) was added to the cell line cultures to prevent mycoplasma contamination. Cell lines were routinely tested for mycoplasma infection by PCR and cell line identity was verified by using the AmpFISTR identifier kit (Thermo Fisher Scientific).

### Retroviral transfection of FL cells

Supernatants of Phoenix cells containing retroviral particles[1] were used to transduce WSU-FSCCL or RL cells by centrifugation (2500 rpm for 90 minutes) in the presence of polybrene (Sigma Aldrich). Selection of transfected cells was started 2 days after infection with 200 µg/mL of hygromycin B (Sigma Aldrich). The Luciferase Assay System kit (Promega) was used to confirm expression of the luciferase gene, following the manufacturer’s instructions.

**Monocyte migration assay**

Monocytes were isolated from freshly isolated PBMCs as described in main methods by RosetteSep. Monocytes were challenged to migrate for 1.5 hours towards cell culture supernatants. LIVE/DEAD® fixable Aqua dead cell stain (Thermo Fisher Scientific) was added 1:100 in PBS to exclude dead cells. The number of viable monocytes ($CD14^+$, $Aqua^-$) was counted in a flow cytometer.

**RNA isolation and quantitative real-time PCR**

For total RNA isolation, 3–5 × $10^6$ cells were homogenized with TRIzol reagent (Thermo Fisher Scientific), following the manufacturer's protocol. cDNA was synthesized using PrimeScript™ RT Master Mix (Takara, Saint- Germain-en-Laye, France) and quantitative real-time PCR (qPCR) was then performed on a StepOne Real-Time PCR System (Thermo Fisher Scientific). Premix Ex Taq (Probe qPCR) Master Mix (Takara) and pre-designed TaqMan® Probes (Thermo Fisher Scientific) for *PPARG, TGFB, MRC1, CCL5, CCR7* and *CXCL11* genes were used. The comparative cycle threshold method (ΔΔCt) was used to quantify the relative expression of each gene. *GUS* was used as endogenous control and samples were analyzed in duplicates.

**Gene expression profiling (GEP) and data analysis**

FL cells from monocultures or co-cultures were purified using CD20 magnetic beads (Miltenyi, Bergisch Gladbach, Germany). Then, total RNA was obtained using the TRIzol reagent (Thermo Fisher Scientific) followed by a cleaning step with the RNAeasy kit (Thermo Fisher Scientific). RNA integrity was examined with the Agilent 2100 Bioanalyzer (Agilent Technologies, Santa Clara CA, USA). Only high-quality RNA was then retrotranscribed to cDNA and hybridized on HG-U219 microarray. All samples were simultaneously run in a GeneTITAN platform (Thermo Fisher Scientific). Data was normalized to RMA using Expression Console software (Thermo Fisher Scientific). GeneChip Command Console Software was used for the scanning of the

results. Raw data were normalized using Expression Console™ Software v1.4.1.46 (Affymetrix). Data has been deposited at the Gene Expression Omnibus (GEO) of the National Center for Biotechnology Information (GSE147211).

Gene signatures were determined with GSEA version 4.0.1 (Broad Institute, Cambridge, MA, USA) using the hallmark gene sets, the C2 curated gene sets, the C3 motif gene sets, the C5 gene ontology gene sets, the C6 oncogenic signatures (Molecular Signature Database v2.5), and custom gene sets (www.lymphochip.com). A two-class analysis with 1000 permutations of gene sets and a weighted metric was used. Expression heatmaps of angiogenesis, integrin pathways, invasion, migration and cytokine genes were created using Morpheus software (https://software.broadinstitute.org/morpheus/, Broad Institute)

Fold-changes of FL-M2 co-cultures *vs* FL monocultures and P-value criteria were represented in a volcano plot. Data were visualized with the Multiplot Studio V1.5.20 Software from GenePattern archive (http://gparc.org/). Top 177 overexpressed genes selected from volcano plot were uploaded to PANTHER (http://www.pantherdb.org/) and subjected to a Mann–Whitney U-test to identify enrichment of PANTHER pathways.

**Bioinformatics analysis**

mRNA relative expression levels of *CSF1* and *CSF1R* in Follicular lymphoma lymph nodes (FL-LN = 362) was compared with normal tonsils (NTS = 75) according to GEP public databases (GSE55267, GSE65135, GSE65136, GSE7307, GSE71810, GSE12195, GSE12366, GSE12453, GSE99316, GSE39503, GSE38712, GSE31311, GSE15271, GSE10831, GSE3526, GSE21554, GSE53820, GSE12453, GSE93261). These selected data were all generated with Affymetrix Human Genome U133 Plus 2.0. Briefly, CEL files were normalized using the Expression Console ™ Software v1.4.1.46 (Affymetrix). To take in consideration the batch effect, join data were normalized using the Limma package included in Transcriptome Analysis console (Applied Biosystems).

CIBERSORT algorithm was used to estimate the proportion of infiltrating immune cell types in gene expression data.[2] This is a robust and accurate algorithm web tool to calculate the cell composition of a given sample. Gene expression data was uploaded to the CIBERSORT website (http://cibersort.stanford.edu/), applying the LM22 signature and 1000 permutations. For each sample, CIBERSORT output was normalized to 100 for each cell type.

**Flow cytometry**

Surface marker analysis was performed on an Attune flow cytometer and the data were analyzed using Attune software (Thermo Fisher Scientific) and FlowJo V10 (FlowJo, LLC, Ashland, OR). The following antibodies were used: CD19-FITC, CD14-PE, CD20-FITC, CD3-PE-Cy5, CD163-FITC, CSF-1R-BV421 and CD49d-PE-Cy5 from BD biosciences, and CD19-eFluor450, CD20-eFluor450, CD3-FITC, CD11b-PE-Cy5, CD18-PE, CD29-FITC and CD86-PE from Thermo Fisher Scientific.

For CSF-1R intracellular staining, cells were fix with 4% paraformaldehyde for 10 min and permeabilized with 0.1% saponin in 0.5% bovine serum albumin/phosphate-buffered saline (PBS) buffer for 5 min.

Viability of FL cell lines and patient samples in monocultures, co-cultures or after treatment with PLX3397 (Selleckchem), was assessed either by CD20 or CD19/Annexin-FITC/7AAD staining or by CD19/CD20, AQUA staining. FL viable cells were identified as $CD19^+/CD20^+$, and $AQUA^-$ or $Annexin\text{-}FITC^-/7AAD^-$.

Monocyte and macrophages were identified with CD11b or CD14 and the expression of specific markers CD86, CD163, CSF-1R, CD18, CD29, CD49d was analyzed. Prior to antibody addition, monocyte/macrophages were treated with FcR Blocking Reagent (Milteny).

MFIR (mean fluorescence intensity ratio) was calculated as follows:

$$MFIR\ (mean\ fluorescence\ intensity)\ = \frac{MFI\ (marker)}{MFI\ (isotype)}$$

**MTT assay**

FL cell lines (5 x $10^4$) or macrophages (2.5 x $10^4$) were incubated for 72 hours with 50nM PLX3397. MTT (3-(4,5-dimethylthiazolyl-2)-2,5-diphenyltetrazolium bromide) reagent (Sigma) was added for 2 to 5 additional hours before spectrophotometric measurement. Each measurement was made in triplicate, and the mean value was calculated. Results were represented using untreated control cells as a reference.

**Cytokine secretion**

CCL2 expression from FL and FL+HK culture supernatants was assessed by Human MCP-1 (CCL2) ELISA kit (ELH-MCP1-1, RayBiotech) following manufacturer´s instructions.

VEGF-A, Angiogenin, MMP9, SDF1-beta, CCL2, CCL3, CCL18, IL-10 and IL-8 secretion were evaluated in culture supernatants (SUPS) from FL and FL+M2 primary co-cultures using a Custom Quantibody® Multiplex ELISA Array (Raybiotech).

CSF-1 expression was evaluated in patient's serum and cell culture supernatants from FL+M2 co-cultures w/wo PLX3397 using RayBio® Human M-CSF ELISA Kit (ELH-MCSF-1, RayBiotech) and following manufacturer´s instructions. IL-10 and IL-34 were also tested in those cell culture supernatants using ABTS ELISA Buffer Kit with IL-10 (#200-10) IL-34 (#200-34) antibodies from Peprotech and following manufacturer´s instructions.

**HUVEC tube formation assay**

Human umbilical vein endothelial cells (HUVEC) were kindly provided by Dr Maria C. Cid and were cultured as previously described.[3] Supernatants from FL primary cells (2x$10^6$ cells/mL) were harvested after 48 hours from FL monocultures or FL+M2 macrophages co-cultures. 24-well plates were coated with Matrigel (BD Biosciences, San Jose, CA, USA) and allowed to polymerize for 45 minutes at 37ºC. Afterwards, the supernatants of interest were mixed (1:1) with HUVEC cells (4x$10^4$ cells) in HUVEC medium (RPMI 1640 medium that contains 25% of

Bovine Calf Serum (HyClone, Thermo Fisher Scientific, Waltham, MA, USA), 150 µg/ml medium endothelial cell growth supplement (ECGS) (BD Bioscience), and 4U/ml medium of sodium heparin salt (AppliChem, Darmstadt, Germany) and incubated for 24 hours. Pictures were taken at 400x magnification in a DMIL LED Leica microscope coupled to a DFC295 camera and analyzed with Suite v 3.7 software (Leica, Wetzlar, Germany). Then, the number of nodes and junctions was quantified in 5 randomly chosen fields using the Image J software (angiogenesis analyzer plugin).

**Adhesion assay to extracellular matrix proteins**

FL cells cultured w/wo M2 macrophages for 48 hours were recovered, counted and labeled with 1 µM Calcein AM (Thermo Fisher Scientific) for 30 minutes at 37ºC. After washing twice with PBS, $1x10^5$ cells/well were seeded in a plate previously coated with 500 ng/mL VCAM-1 (R&D Systems), 10 mg/mL fibronectin (Millipore) or BSA 1% (nonspecific adhesion) and incubated overnight at 4C. Then, the plate was washed repeatedly washed to remove non-adhered cells. Adhered cells were lysed with 1% Triton X-100, supernatant was transferred into black plates and fluorescence was measured in a spectrophotometer (Synergy Bio-Tek Instrument) (excitation filter: 485 ± 20 nm; band-pass filter: 530 ± 20 nm). Data were expressed as arbitrary fluorescent units after subtraction of nonspecific adhesion from VCAM1/fibronectin adhesion and normalized to the untreated control.

**Invasion assay**

FL cells co-cultured w/wo macrophages in a ratio (1:4) for 48 hours, were seeded ($0.5x10^6$cells/well) on Matrigel Invasion Chambers 8µm (BD Biosciences) in RPMI 10% FBS, and were allowed to migrate in a gradient of FBS (20%) for 24 hours. Cells crossing the barrier were labeled with anti-CD20-FITC and counted by flow cytometry. Migration ratio was calculated as the ratio between the number of $CD20^+$ in each condition and the corresponding input.

***In vivo* studies**

An FL-HK mouse model was generated by subcutaneous inoculation of the FL cell lines WSU-FSCCL or RL (10 x $10^6$ cells per mouse) with/without HK cells (1x$10^6$ cells per mouse, ratio HK:FL 1:10) in severe combined immunodeficient (SCID) mice. Tumors were measured twice weekly with external calipers. Tumor volumes were calculated by the formula:

$$Tumor\ volume = (Tumor\ width^2 x\ Tumor\ length)/2$$

Each graph shows mean ± SEM. At the endpoint, tumors were extirpated, weighed, formalin-fixed and included in paraffin (FFPE). Animals who did not develop tumor were excluded.

For macrophage depletion studies two types of experiments were done

1) WSU–FSCCL cells (10x$10^6$ cells per mouse) were admixed with the FDC non-immortalized cell line HK (1x$10^6$ cells per mouse) and subcutaneously inoculated into the right flank of SCID mice. Tumor-bearing animals were randomized by tumor size into two groups (n = 10/group) for intratumoral injection of 50 µL of clodronate liposomes (Liposome concentration = 5mg/mL) or an equal volume of empty control liposomes, 2 days per week for 14 days. Tumor follow-up and volume calculation were done described in the main methods.

2) WSU–FSCCL cells (10x$10^6$ cells per mouse) were intravenously inoculated into NOD SCID NSG (NOD SCID gamma null) mice *via* tail vein. Six days later, they were randomized by mouse weight into two groups by weight (n = 6-7/group) and treated *i.v.* with 25 µL of clodronate liposomes (Liposome concentration = 5mg/mL) or an equal volume of control empty liposomes, once a week, during 20 days (ClodronateLiposomes.org, Haarlem, the Netherlands). Luciferase activity, reported as Relative Luminescence Units (RLU), was measured and monitored on a Hamamatsu ORCA II digital camera before treatment (Day 18) and one day before the end point (Day 28). At the end point spleens were extirpated, weighed, formalin-fixed and included in paraffin (FFPE).

For the combo experiments, a FL-HK mouse model was generated by subcutaneous inoculation of the FL cell line RL-luciferase (10 x $10^6$ cells per mouse) with HK cells (1x$10^6$ cells per mouse,

ratio FDC:FL 1:10) in SCID mice (n=6 mice per group). Treatments started when tumor volumes reached 200 $mm^3$ (Day 16). Mice were treated for 14 days w/wo PLX3397 (50 mg/kg/day, p.o.), and/or Rituximab (20-10-10 mg/kg mouse, i.p.) once a week.

Animal experiments were performed in compliance with the Animal Ethics Committee of the University of Barcelona (agreements #89/18 and 90/18).

**Immunohistochemistry**

FFPE sections from representative FL and FL+HK tumors w/wo treatments, and spleens from clodronate experiments were stained with the following antibodies: the pan-macrophage marker F4/80 (clone Cl:A3-1, Bio-Rad), the M2-marker CD206 (clone MMR, R&DSystems), the M1-marker iNOS (ab15323, Abcam), Phospho-histone H3 (pH3) (phospho S10 + T11 antibody [E173], ab32107, Abcam), CD10 (clone SP67, Roche) and CD20 (clone L26, Roche) and PECAM-1 (clone M-20, Santa Cruz Biotechnology). Automatic immunostaining was performed using the Leica Microsystems' Bond-max™ together with the Bond Polymer Refine Detection System (Leica Microsystems, Spain). Slides were scanned and image processing was done using Pannoramic Viewer (3DHISTECH Ltd) and Olympus CellSens 1.17 (Olympus America Inc.) software. Quantification and analysis were done in the using ImageJ software (magnification 200x). % of mitotic cells was calculated on a percentage basis according to the following equation:

$$\%\ of\ mitotic\ cells = \frac{pH3\ positive\ cells\ per\ field}{Blue\ nuclei\ (Hematoxylin)\ per\ field} \times 100$$

FFPE sections of FL patients from Hospital Clínic were probed for CSF-1R expression using the CSF-1R antibody clone FER216 developed at the Monoclonal Antibodies Unit of CNIO and kindly provided by Dra. Giovanna Roncador, using the Leica automatic immunostainer as above. The staining was visualized using an upright optical microscope (Olympus BX63) and after digitalization with a slide scanner (Nanozoomer S360, Hamamatsu), the digital

quantification of CSF-1R expression was done with Fiji-Image J software as previously described.[4]

**Statistical analysis**

Depending on the assay, paired t test or Mann-Whitney test were used. For *in vivo* tumor growth experiments the multiple t-Tests (Holm-Sidak method) was used to correct for multiple comparisons. Outliers were identified with the ROUT method with Q=1%. These analyses were performed using Prism software 7.0. Standard deviation (SD) or Standard Error of Mean (SEM) was calculated among experimental replicates in each case. Shapiro-Wilk test was used to estimate groups variance. Differences between the results of comparative tests were considered significant if the two-sided P value was less than 0.05. The statistical significance convention used along the manuscript was as follows: * p<0.05, **p<0.01 and ***p<0.001.

Kaplan-Meier plot of Progression-Free Survival (PFS) and overall survival (OS) were performed of FL patients grouped on the basis of CSF-1R expression (n = 78). An optimum threshold on the score to separate patients into low and high-expression groups was determined using the MaxStat software to select the cutoff value producing the maximum log-rank score in the training cohort. Cutoff value = 8.335 for follicular Compartment, and 21.379 for interfollicular. P-value was obtained by the overall log-rank test.

The correlation of CSF-1R with clinical variables (Categorical data) was done using Chi square or Fisher's exact test with two-sided p values using SPSS 22. Non-parametric tests were used when necessary. To estimate the risk of HT, where competitive events are possible, cumulative incidence was calculated (cmprsk R package) and compared with Gray's test. Actuarial survival analysis was performed using Kaplan–Meier, and survival curves were compared using the log-rank test. p values<0.05 were considered statistically significant.

SUPPLEMENTAL FIGURES

# Figure S1

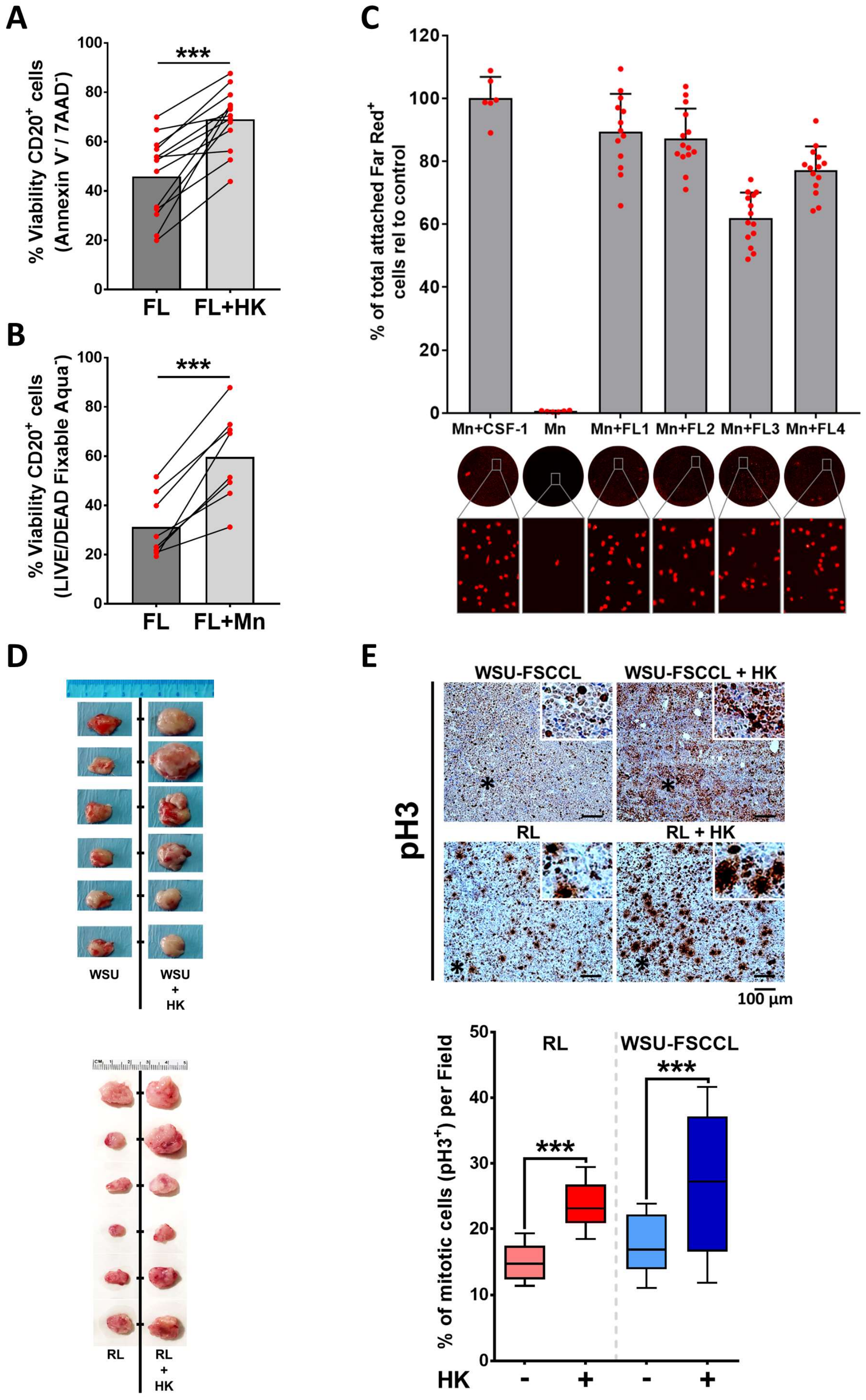


**Figure S1. FL-FDC and FL-monocyte crosstalk. (A)** Viability of FL cells (CD20+/Annexin V-/7AAD-) in monocultures and in FL-FDC co-cultures (48h, n=13) Paired *t* test. **(B)** Viability of FL cells (CD20+/AQUA-) in monocultures and FL-monocytes (Mn) co-cultures (n=13) after 48h assessed by flow cytometry cells. Paired *t* test. **(C)** Monocyte differentiation assay was assessed co-culturing FL primary cells (n = 4) with monocytes from PBMC (ratio 4:1) labeled with CellTrace™ Far Red for 7 days. Number of attached macrophages (Far Red+) was analyzed by cell counting using Cytation™ 1 Cell Imaging Multi-Mode Reader (BioTek). **(D)** Pictures from FL-FDC co-xenografts shown in figure 1D. **(E)** Phospho-histone H3 (pH3) staining by IHC in tumors from S1D. Representative images are shown. Quantification and analysis were done using ImageJ software (magnification 200x) as detailed in the supplemental methods section. Mann-Whitney test.

## Figure S2

**A**

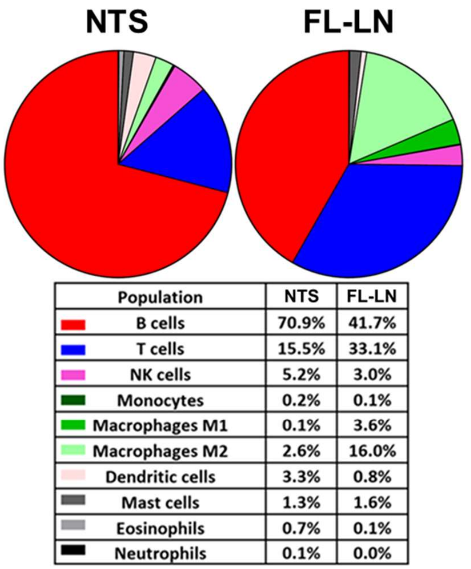


| Population | NTS | FL-LN |
|---|---|---|
| B cells | 70.9% | 41.7% |
| T cells | 15.5% | 33.1% |
| NK cells | 5.2% | 3.0% |
| Monocytes | 0.2% | 0.1% |
| Macrophages M1 | 0.1% | 3.6% |
| Macrophages M2 | 2.6% | 16.0% |
| Dendritic cells | 3.3% | 0.8% |
| Mast cells | 1.3% | 1.6% |
| Eosinophils | 0.7% | 0.1% |
| Neutrophils | 0.1% | 0.0% |

**B**

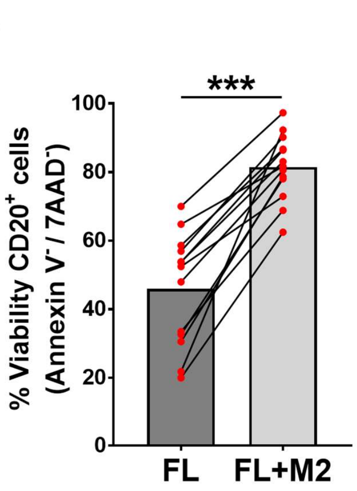


**C**

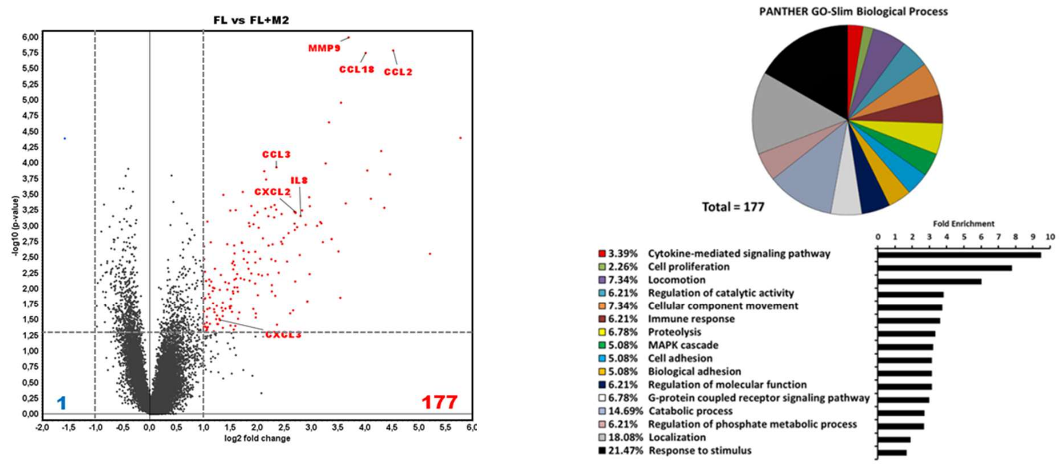


**D**

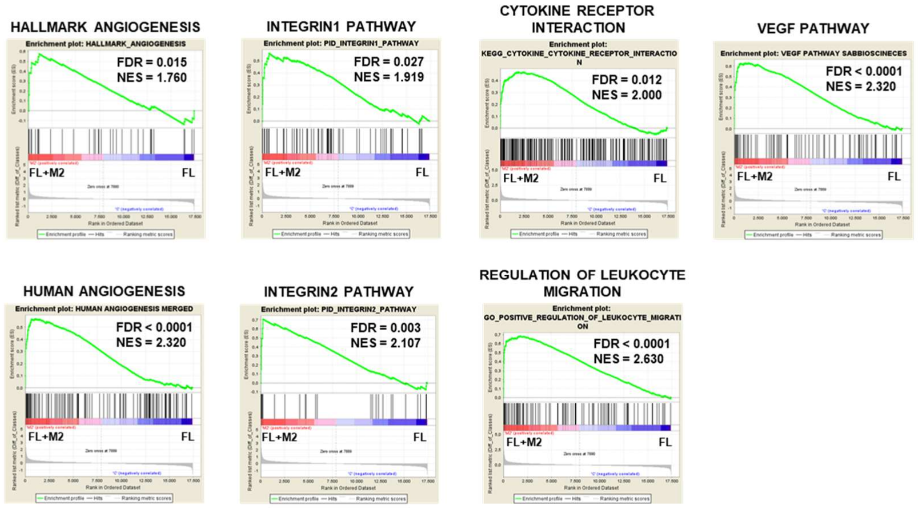


**Figure S2. FL-LN are enriched in M2 macrophages and contribute to FL tumorigenesis. (A)** Composition of infiltrating immune cells in Normal Tonsils (NTS, n = 75) or Follicular Lymphoma Lymph nodes (FL-LN, n = 362) evaluated by CIBERSORT in Affymetrix datasets and summarized from calculated mean values for each group. **(B)** Viability of FL cells ($CD20^+$/Annexin $V^-$/$7AAD^-$) in monocultures and FL-M2 co-cultures (48h, n=13) Paired *t* test. Bars represent the SD. **(C)** Volcano plot for FL monocultures vs FL-M2 co-cultures comparison (Left panel). -log10 P-value is plotted against the fold change (log2 scale). Black horizontal discontinuous line corresponds to a P-value of 0.05. 177 genes with a P-value < 0.05 and fold change>2 in either direction are labeled (Red (177 upregulated) and blue (1 downregulated)). Enrichment of biological processes found by PANTHER gene ontology analysis of the 177 volcano-filtered genes overexpressed in FL-M2 co-cultures (Right panel). **(D)** Representative enrichment plots of adhesion, invasion, angiogenesis and cytokine secretion gene sets are shown. FDR: false discovery rate. NES: normalized enrichment score.

# Figure S3

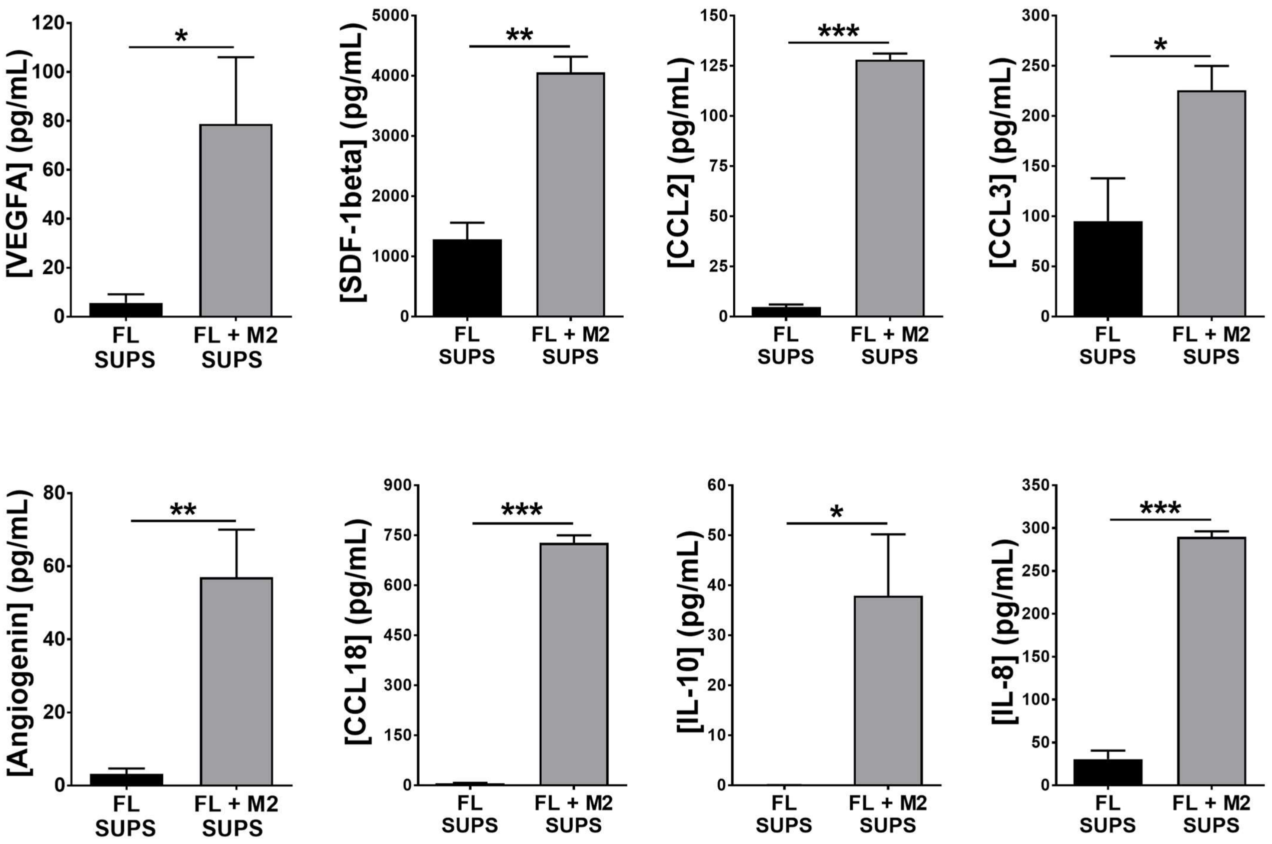


**Figure S3. M2 macrophages increase FL cells cytokine secretion.** Supernatants from the FL or FL-M2 co-cultures described in Figure 2 were assayed for the cytokines indicated related to the processes identified by GEP. Statistical significance was analyzed by paired t test. Bars represent the SD.

## Figure S4

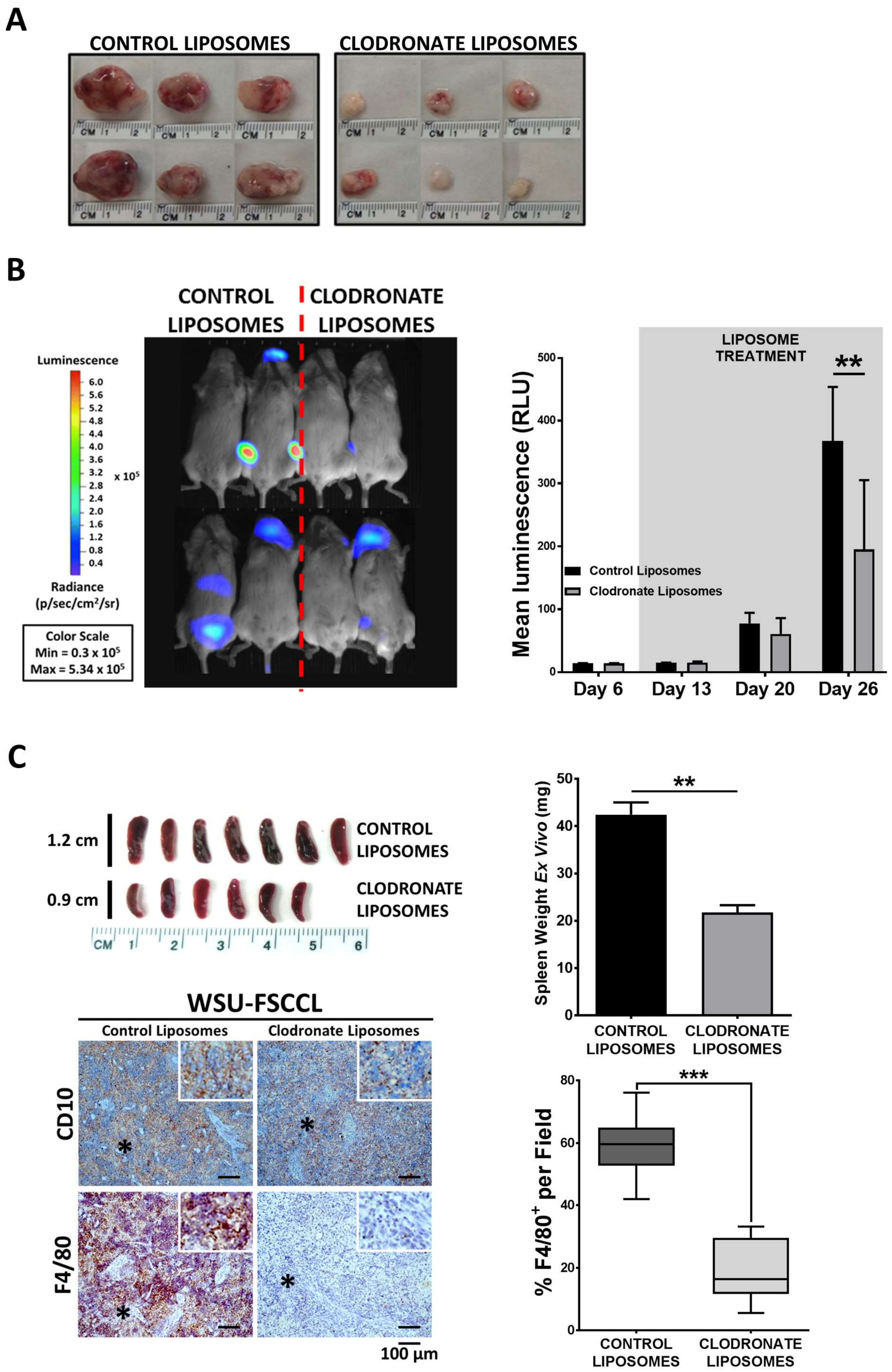


**Figure S4. Macrophage depletion with liposomal clodronate in an intravenous mouse model of FL. (A)** Representative images of the collected tumors from the subcutaneous model are shown of each treatment group. **(B)** WSU–FSCCL cells ($10x10^6$ cells per mouse) were intravenously inoculated into NSG mice at day 0, and then randomized and treated at day 7 for clodronate liposomes or an equal volume of control empty liposomes, once a week, during 19 days. Luciferase activity, reported as Relative Luminescence Units (RLU), was measured and monitored weekly. Bar-graph shows mean ± SEM. Statistical significance was analyzed by multiple t-Tests (Holm-Sidak method). **(C)** Spleens from S4B were extirpated, weighed and measured at the end point. Mann-Whitney test was applied. FFPE sections from spleens were stained with the germinal center marker CD10 and the pan-macrophage marker F4/80 (magnification 200x). Representative images are shown (left panel). Quantification was done using ImageJ software (right panel). Mann-Whitney test. Bars represent the SD.

# Figure S5

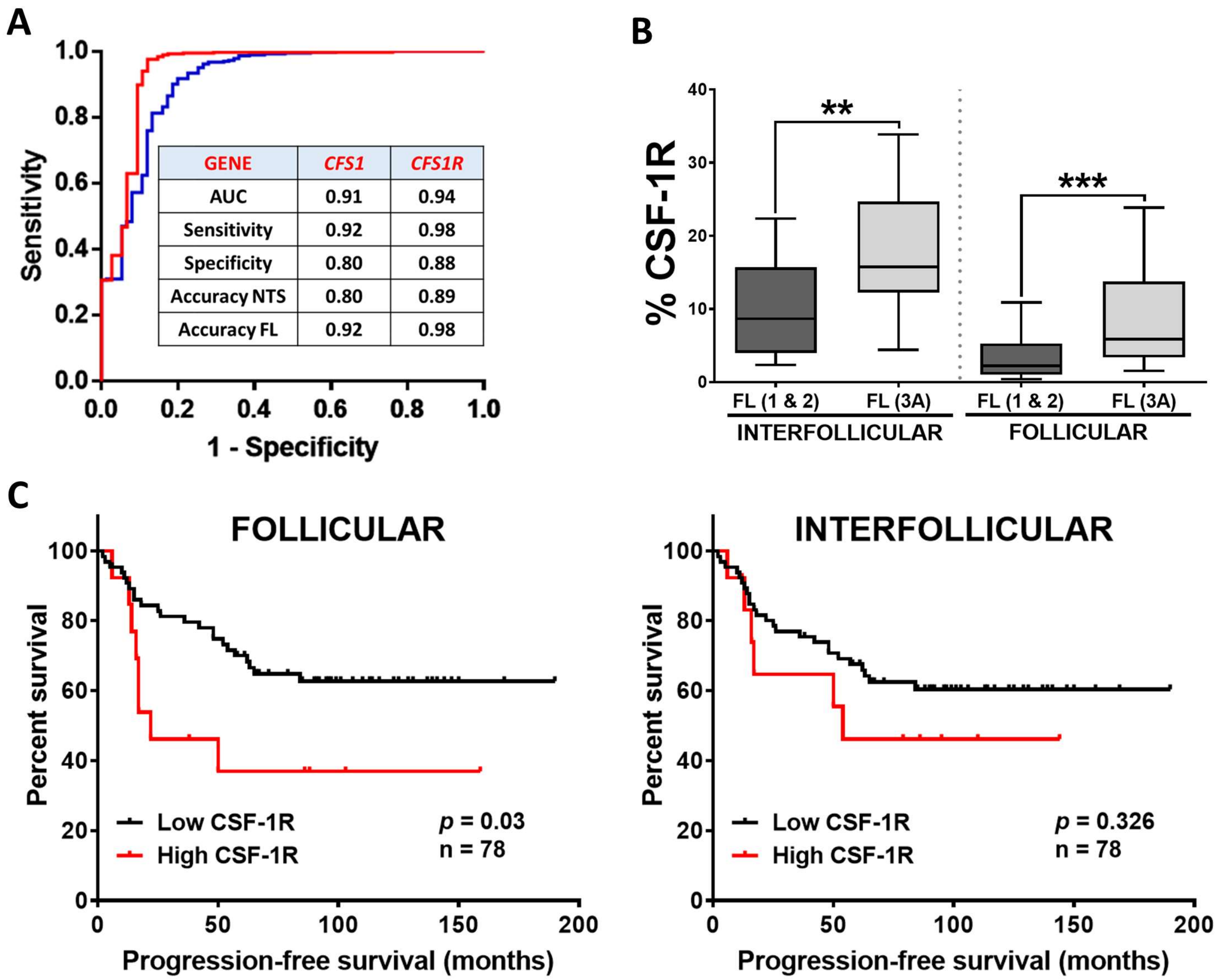


**Figure S5. CSF-1 and CSF-1R expression in FL and prognostic value. (A)** ROC curves of *CSF1* and *CSF1R* mRNA expression with the significantly highest AUC (Area Under Curve) calculated from the comparison between NTS and FL-LN groups of Figure 5A. **(B)** Comparison of follicular and interfollicular CSF-1R expression (FL series from Figure 5C, n=78) between grade 1-2 (56) and Grade 3A (22) FL patients. Statistical significance was analyzed by Mann-Whitney test. **(C)** Kaplan-Meier plot of Progression-free survival of FL patients grouped on the basis of CSF-1R expression. An optimum threshold on the score to separate patients into low and high-expression groups was determined using the MaxStat software to select the cutoff value producing the maximum log-rank score in the training cohort. Cutoff value = 8.335 for follicular Compartment (left panel), and 21.379 for interfollicular (right panel). *P*-value was obtained using the overall log-rank test.

# Figure S6

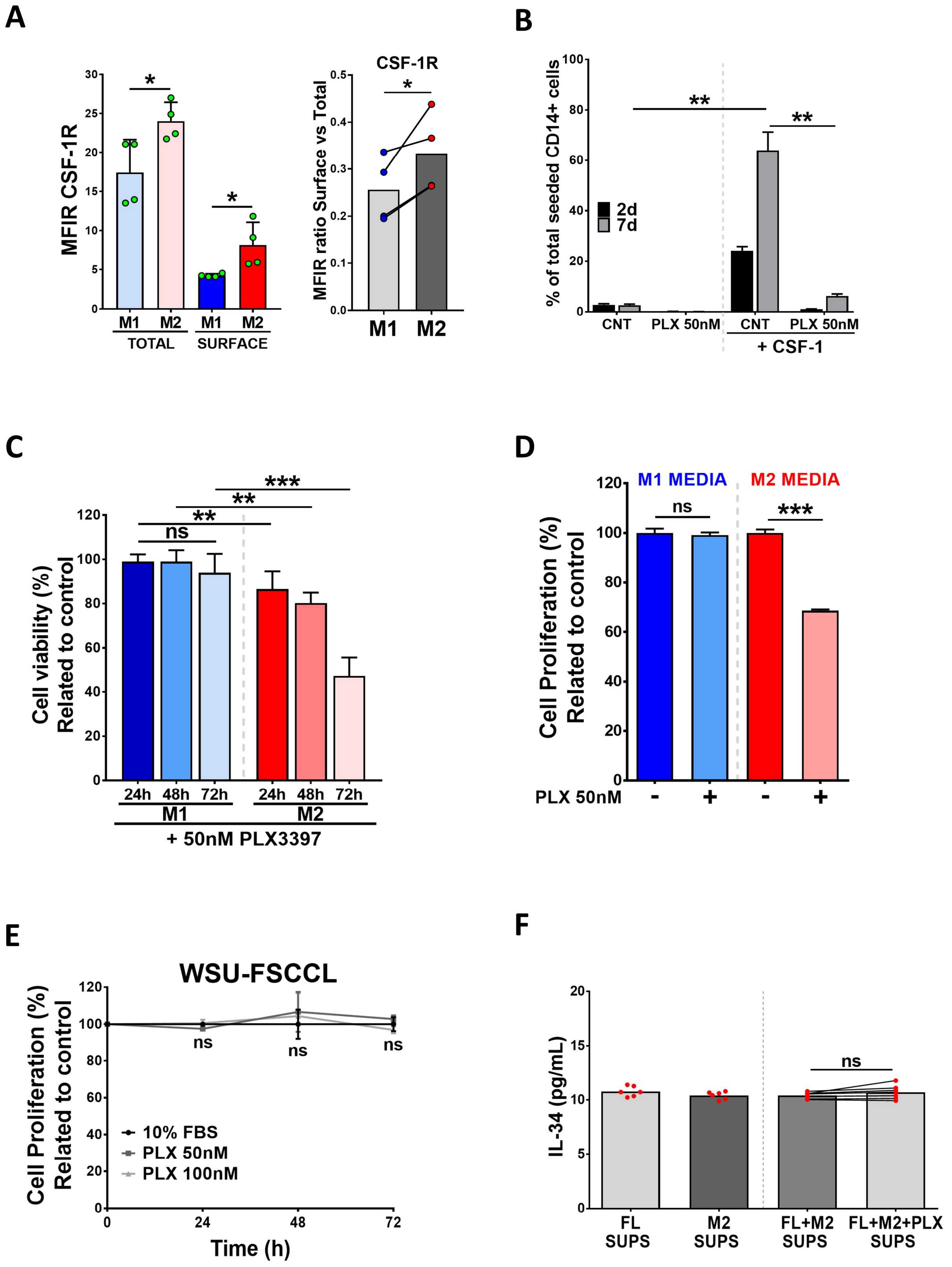


**Figure S6. Effect of CSF-1R targeting with PLX3397 in macrophages and FL cells. (A)** Surface and total CSF-1R expression was analyzed by flow cytometry in M1 (CSF-1 + IFNγ + LPS) and M2 (CSF-1 + IL4)-polarized macrophages. MFIR was calculated as follows: MFIR (mean fluorescence intensity ratio) = MFI (marker)/MFI (isotype). MFIR ratio between CSF-1R surface and total expression from Figure S6A was calculated as MFIR (surface)/ MFIR (total). Paired t test (**B)** Human monocytes (5x$10^5$ cells) from PBMCs were pre-treated for 1h with 50nM PLX3397 and then seeded in the presence or absence of CSF-1. Macrophage count was assessed by flow cytometry (CD14+, AQUA- cells) at day 2 and 7 of treatment. Paired t test **(C)** MTT assay of M1 or M2 macrophages in the presence or absence of the CSF-1R inhibitor PLX3397 for the times indicated. **(D)** WSU-FSCCL cells were plated with medium from M1 or M2 macrophage cultures (n=6) treated or not with PLX3397 (50nM). Viability was assessed by MTT assay. Results are expressed as % of control and represent the mean ±SD of 6 donors. Paired t test. **(E)** WSU-FSCLL was treated with PLX3397 and viability was assessed by MTT assay at the times indicated. Multiple t-Tests (Holm-Sidak method). **(F)** IL-34 was detected by ELISA in supernatants from Figure 6E. Paired t test.

# Figure S7

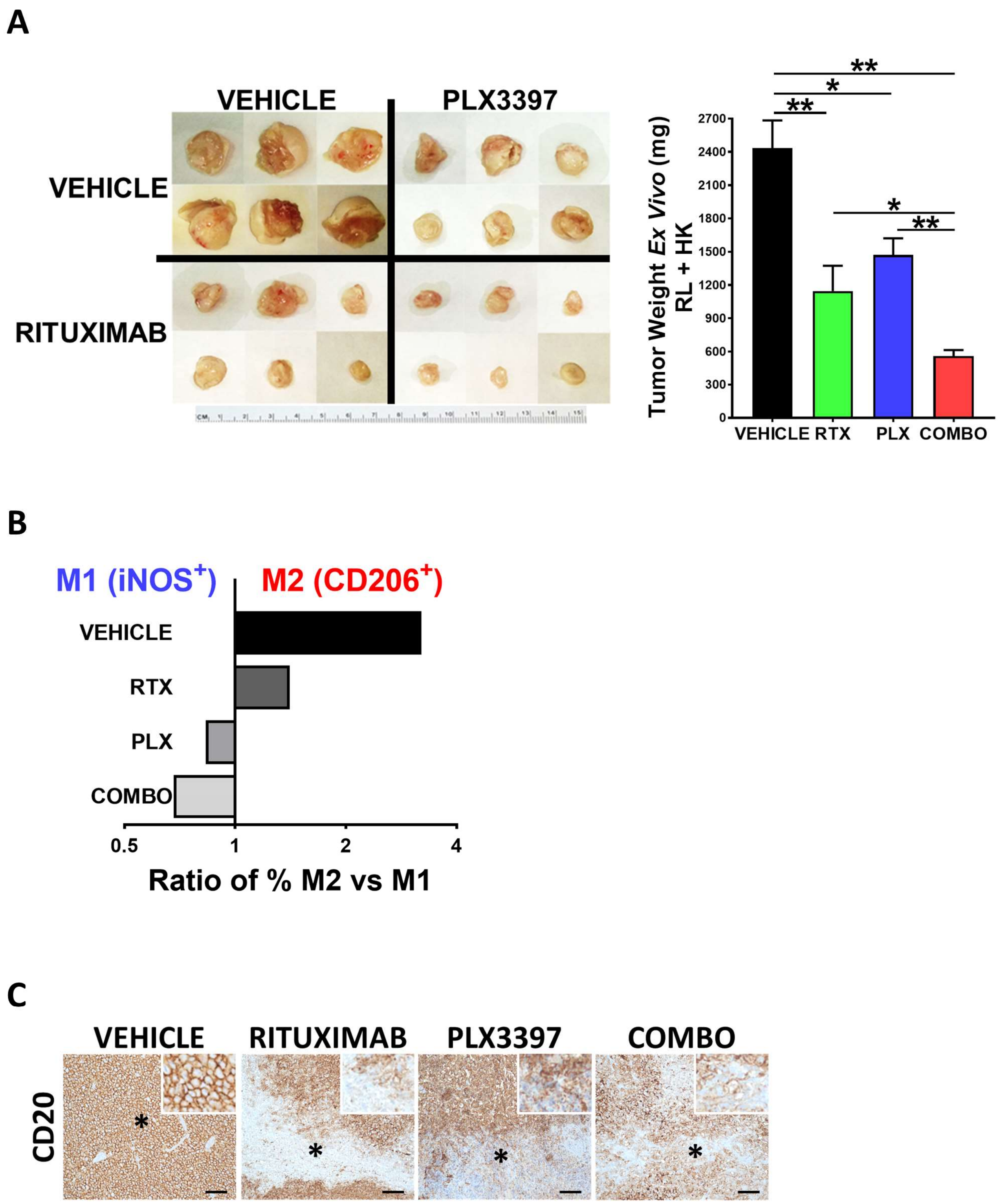


**Figure S7.** ***In vivo*** **PLX3397 and rituximab combo complementary results. (A)** Tumor were excised and weighted at the endpoint. Representative images are shown and graph shows tumor and weight at the endpoint where mean ± SEM are plotted the multiple t-Tests (Holm-Sidak method). **(B)** Effect of PLX3397 on M2/M1 ratio from figure 7B. **(C)** CD20 expression from Figure 7B.

**SUPPLEMENTAL TABLES**

**Table S1. FL patients (*in vitro + serums*)**

| STUDY LABEL | GENDER[1] | AGE | SAMPLE TYPE[2] | % TUMOR CELLS[3] | HISTOLOGIC GRADE[4] | STAGE[5] | FLIPI[6] | DISEASE STATUS[7] |
|---|---|---|---|---|---|---|---|---|
| FL01 | M | 71 | LN | 49 | 2 | II | 2 | D |
| FL02 | M | 77 | LN | 30 | NA | NA | NA | NA |
| FL03 | F | 83 | LN | 75 | 2 | III | 2 | D |
| FL04 | M | 65 | LN | 75 | 2 | I | 1 | D |
| FL05 | F | 73 | LN | 73 | 2 | IV | 5 | D |
| FL06 | M | 80 | LN | 85 | 2 | IV | 3 | D |
| FL07 | F | 52 | LN | 85 | 1 | IV | 3 | D |
| FL08 | F | 78 | TS | 36 | 3B | III | 2 | D |
| FL09 | F | 75 | PB | 78 | 2 | IV | 4 | D |
| FL10 | M | 56 | LN | 33 | 3B | IV | 2 | D |
| FL11 | M | 50 | LN | 68 | 1 | IV | 2 | D |
| FL12 | M | 65 | LN | 37 | 2 | IV | 2 | D |
| FL13 | M | 52 | LN | 78 | 2 | IV | 3 | D |
| FL14 | F | 66 | PM | 52 | 1 | IV | 2 | D |
| FL15 | M | 63 | PB | NA | 2 | IV | 2 | D |
| FL16 | F | NA | LN | 75 | NA | NA | NA | NA |
| FL17 | F | 52 | LN | 85 | 1 | IV | 2 | D |
| FL18 | M | 75 | LN | 75 | 2 | I | 2 | D |
| FL19 | M | 70 | PB | 79 | 2 | IV | 3 | D |
| FL20 | M | 64 | S | N/A | 1 | IV | 2 | D |
| FL21 | F | 56 | S | N/A | 1 | IV | 1 | D |
| FL22 | M | 45 | S | N/A | 2 | I | 0 | D |
| FL23 | F | 54 | S | N/A | 2 | IV | 2 | D |
| FL24 | F | 54 | S | N/A | 2 | II | 0 | D |
| FL25 | F | 61 | S | N/A | 2 | III | 2 | D |
| FL26 | M | 35 | S | N/A | 2 | IV | 3 | D |
| FL27 | F | 65 | S | N/A | 2 | IV | 3 | D |
| FL28 | F | 51 | S | N/A | 2 | IV | 3 | D |
| FL29 | F | 52 | S | N/A | 2 | I | 0 | D |
| FL30 | M | 56 | S | N/A | 2 | IV | 2 | D |
| FL31 | M | 42 | S | N/A | 2/3 | IV | 2 | D |
| FL32 | M | 74 | S | N/A | 3A | III | 2 | D |
| FL33 | M | 42 | S | N/A | 3A | IV | 3 | D |
| FL34 | F | 65 | S | N/A | 3A | IV | 3 | D |
| FL35 | M | 54 | S | N/A | 3A | IV | 2 | D |
| FL36 | M | 72 | S | N/A | 3A | III | 2 | D |

[1] F:Female; M:Male; [2] PB: Peripheral Blood, LN: Lymph Node, TS: Tonsil, PM: Paravertebral Mass, S: serum; [3] % of cells positive for CD20, CD10 and showing light chain restriction; [4] Grade was evaluated by two different pathologists; [5]Ann Arbor stage; [6]FLIPI: Follicular Lymphoma International Prognostic Index; [7]Samples were obtained at D: diagnosis, R: relapse, NA: Not Available, N/A: Not Applicable

**Table S2. Gene sets overrepresented in FL cells after co-culture with M2 macrophages**

| GENE SET NAME | # of enriched gene sets | NES (max) | FDR, q value (min) |
|---|---|---|---|
| **Custom gene sets** | | | |
| Immune response 2 FL bad prognosis | 1 | 2.40 | <0.0001 |
| Angiogenesis | 3 | 2.32 | <0.0001 |
| Serum response | 1 | 2.17 | 0.0001 |
| NF-kB pathway | 2 | 1.99 | 0.0001 |
| Toll Like Receptors (TLRs) | 1 | 1.87 | 0.0130 |
| **Canonical pathways gene sets (C2CP)** | | | |
| Toll Like Receptors (TLRs) | 3 | 2.32 | <0.0001 |
| Antigen presentation | 2 | 2.28 | 0.0002 |
| Chemokines and chemokine receptors | 3 | 2.26 | 0.0002 |
| IL8 pathway | 2 | 2.14 | 0.0022 |
| Interferon gamma signaling | 1 | 2.13 | 0.0022 |
| Integrin pathway | 4 | 2.11 | 0.0032 |
| Extracellular matrix (ECM) regulation | 3 | 2.09 | 0.0039 |
| Cytokines and cytokine receptors | 2 | 2.00 | 0.0120 |
| CCR5 pathway | 1 | 1.98 | 0.0147 |
| Syndecan 2,4 pathway | 2 | 1.97 | 0.0185 |
| **Motif gene sets (C3)** | | | |
| V$STAT3_01 | 1 | 1.97 | 0.0203 |
| **Hallmark gene sets (H)** | | | |
| KRAS signaling | 1 | 2.47 | <0.0001 |
| IL6 -JAK-STAT3 signaling | 1 | 2.39 | <0.0001 |
| Inflammatory response | 1 | 2.27 | <0.0001 |
| TNFalpha-NF-kB signaling | 1 | 2.21 | 0.0002 |
| Interferon gamma response | 1 | 2.00 | 0.0009 |
| Epithelial mesenchymal transition | 1 | 1.98 | 0.0013 |
| IL2-STAT5 signaling | 1 | 1.89 | 0.0021 |
| Angiogenesis | 1 | 1.77 | 0.0149 |
| **GO gene sets (C5)** | | | |
| Leukocyte chemotaxis | 23 | 2.63 | <0.0001 |
| Interferon gamma response | 3 | 2.49 | <0.0001 |
| Toll Like Receptors (TLRs) | 5 | 2.46 | <0.0001 |
| TNFalpha | 4 | 2.45 | <0.0001 |
| Inflammatory response | 4 | 2.45 | <0.0001 |
| Chemokine signaling | 7 | 2.41 | <0.0001 |
| Regulation of endocytosis | 4 | 2.35 | <0.0001 |
| Extracellular matrix disassembly | 2 | 2.39 | <0.0001 |
| IL6 synthesis | 2 | 2.16 | 0.0013 |
| VEGF-VEGFR pathway | 3 | 2.12 | 0.0025 |
| Integrin-mediated cell adhesion | 2 | 2.07 | 0.0043 |
| Antigen processing and presentation | 5 | 2.09 | 0.0034 |
| Angiogenesis-vasculature development | 2 | 1.93 | 0.0193 |
| **Oncogenic gene sets (C6)** | | | |
| KRAS | 10 | 2.260 | <0.0001 |

GSEA was used to test for significant enrichment of defined gene signatures. NES indicates Normalized Enriched Score; FDR, False Discovery Rate. Threshold FDR<0.05 and NES>1.5. Custom genes set were experimentally derived and downloaded from http://lymphochip.nih.gov/signaturedb/index.html. C2CP, C3, C5, C6 and H gene sets were obtained from the Molecular Signature Database (v2.5).

**Table S3. Complete GSEA results**

| CUSTOM GENE SETS | | | | |
|---|---|---|---|---|
| **Biological process** | **Gene set name** | **Size** | **NES** | **FDR q-val** |
| **Immune response 2 FL bad prognosis** | IMMUNE RESPONSE2 FL STAUDTBADPROGNOSIS | 20 | 2.4 | 0 |
| **Angiogenesis** | VEGF PATHWAY SABBIOSCINECES | 80 | 2.32 | 0 |
| | HUMAN ANGIOGENESIS MERGED | 120 | 2.22 | 0.00019 |
| | VEGF PATHWAY TAQMANMICROFLUIDICS | 89 | 1.73 | 0.03165 |
| **Serum response** | SERUM_RESPONSE_FB_UP | 158 | 2.17 | 0.00016 |
| **NF-KB pathway** | NFKB_ALL_OCILY3_LY10 | 54 | 1.99 | 0.00423 |
| | NFKB_OCILY10_ONLY | 15 | 1.72 | 0.03222 |
| **Toll Like Receptors (TLRs)** | TOLLPATHWAY | 30 | 1.87 | 0.01301 |
| **CANONICAL PATHWAYS (C2CP)** | | | | |
| **Biological process** | **Gene set name** | **Size** | **NES** | **FDR q-val** |
| **Toll Like Receptors (TLRs)** | REACTOME_TOLL_RECEPTOR_CASCADES | 104 | 2.32 | 0 |
| | KEGG_TOLL_LIKE_RECEPTOR_SIGNALING_PATHWAY | 90 | 2.13 | 0.00263 |
| | BIOCARTA_TOLL_PATHWAY | 34 | 1.91 | 0.02556 |
| **Antigen presentation** | REACTOME_MHC_CLASS_II_ANTIGEN_PRESENTATION | 78 | 2.28 | 0.00022 |
| | KEGG_ANTIGEN_PROCESSING_AND_PRESENTATION | 63 | 2.1 | 0.00316 |
| **Chemokines and chemokine receptors** | REACTOME_CHEMOKINE_RECEPTORS_BIND_CHEMOKINES | 51 | 2.26 | 0.00018 |
| | KEGG_CHEMOKINE_SIGNALING_PATHWAY | 169 | 2.07 | 0.0055 |
| | PID_CXCR4_PATHWAY | 97 | 1.88 | 0.03381 |
| **IL8 pathway** | PID_IL8_CXCR1_PATHWAY | 28 | 2.14 | 0.00225 |
| | PID_IL8_CXCR2_PATHWAY | 32 | 2.14 | 0.00216 |
| **Interferon gamma signaling** | REACTOME_INTERFERON_GAMMA_SIGNALING | 51 | 2.13 | 0.00253 |
| **Integrin pathway** | PID_INTEGRIN2_PATHWAY | 27 | 2.11 | 0.00322 |
| | PID_INTEGRIN1_PATHWAY | 61 | 1.92 | 0.02677 |
| | PID_INTEGRIN_A4B1_PATHWAY | 30 | 1.92 | 0.02686 |
| | PID_INTEGRIN_A9B1_PATHWAY | 24 | 1.87 | 0.03587 |
| **Extracellular matrix (ECM) regulation** | NABA_ECM_REGULATORS | 214 | 2.09 | 0.00397 |
| | REACTOME_DEGRADATION_OF_THE_EXTRACELLULAR_MATRIX | 26 | 2.05 | 0.00614 |
| | NABA_ECM_AFFILIATED | 142 | 1.92 | 0.02633 |
| **Cytokines and cytokine receptors** | KEGG_CYTOKINE_CYTOKINE_RECEPTOR_INTERACTION | 243 | 2 | 0.01204 |
| | PID_IL23_PATHWAY | 35 | 1.87 | 0.03424 |
| **CCR5 pathway** | BIOCARTA_CCR5_PATHWAY | 16 | 1.98 | 0.01478 |
| **Syndecan 2,4 pathway** | PID_SYNDECAN_4_PATHWAY | 32 | 1.97 | 0.01858 |
| | PID_SYNDECAN_2_PATHWAY | 31 | 1.94 | 0.02341 |
| **MOTIF GENE SETS (C3TFT)** | | | | |
| **Biological process** | **Gene set name** | **Size** | **NES** | **FDR q-val** |
| **STATs** | V$STAT3_01 | 20 | 1.97 | 0.02028 |
| **HALLMARKS (H)** | | | | |
| **Biological process** | **Gene set name** | **Size** | **NES** | **FDR q-val** |
| **KRAS signaling** | HALLMARK_KRAS_SIGNALING_UP | 190 | 2.47 | 0 |
| **IL6 -JAK-STAT3 signaling** | HALLMARK_IL6_JAK_STAT3_SIGNALING | 85 | 2.39 | 0 |
| **Inflammatory response** | HALLMARK_INFLAMMATORY_RESPONSE | 192 | 2.27 | 0 |
| **TNFalpha-NF-kB signaling** | HALLMARK_TNFA_SIGNALING_VIA_NFKB | 192 | 2.21 | 0.00017 |
| **Interferon gamma response** | HALLMARK_INTERFERON_GAMMA_RESPONSE | 178 | 2 | 0.00093 |
| **Epithelial mesenchymal transition** | HALLMARK_EPITHELIAL_MESENCHYMAL_TRANSITION | 189 | 1.98 | 0.00127 |
| **IL2-STAT5 signaling** | HALLMARK_IL2_STAT5_SIGNALING | 184 | 1.89 | 0.00218 |

| | | | | |
|---|---|---|---|---|
| **Angiogenesis** | HALLMARK_ANGIOGENESIS | 35 | 1.77 | 0.00548 |
| **GO GENESETS (C5)** | | | | |
| **Biological process** | **Gene set name** | **Size** | **NES** | **FDR q-val** |
| **Leukocyte chemotaxis** | GO_POSITIVE_REGULATION_OF_LEUKOCYTE_MIGRATION | 102 | 2.63 | 0 |
| | GO_REGULATION_OF_LEUKOCYTE_MIGRATION | 139 | 2.63 | 0 |
| | GO_REGULATION_OF_LEUKOCYTE_CHEMOTAXIS | 89 | 2.59 | 0 |
| | GO_POSITIVE_REGULATION_OF_LEUKOCYTE_CHEMOTAXIS | 75 | 2.58 | 0 |
| | GO_LEUKOCYTE_CHEMOTAXIS | 105 | 2.46 | 0 |
| | GO_MONOCYTE_CHEMOTAXIS | 33 | 2.41 | 0 |
| | GO_REGULATION_OF_GRANULOCYTE_CHEMOTAXIS | 36 | 2.36 | 0 |
| | GO_REGULATION_OF_MONOCYTE_CHEMOTAXIS | 20 | 2.31 | 0.00017 |
| | GO_POSITIVE_REGULATION_OF_CHEMOTAXIS | 112 | 2.31 | 0.00018 |
| | GO_POSITIVE_REGULATION_OF_MONOCYTE_CHEMOTAXIS | 15 | 2.25 | 0.00052 |
| | GO_REGULATION_OF_CHEMOTAXIS | 168 | 2.23 | 0.00068 |
| | GO_REGULATION_OF_MONONUCLEAR_CELL_MIGRATION | 15 | 2.22 | 0.00071 |
| | GO_REGULATION_OF_MACROPHAGE_CHEMOTAXIS | 15 | 2.21 | 0.0008 |
| | GO_REGULATION_OF_NEUTROPHIL_CHEMOTAXIS | 25 | 2.2 | 0.00094 |
| | GO_REGULATION_OF_NEUTROPHIL_MIGRATION | 30 | 2.14 | 0.00203 |
| | GO_LYMPHOCYTE_CHEMOTAXIS | 32 | 2.12 | 0.00264 |
| | GO_POSITIVE_REGULATION_OF_NEUTROPHIL_MIGRATION | 25 | 2.07 | 0.00466 |
| | GO_REGULATION_OF_LYMPHOCYTE_MIGRATION | 35 | 2.05 | 0.00649 |
| | GO_LYMPHOCYTE_MIGRATION | 42 | 1.87 | 0.03222 |
| | GO_NEGATIVE_REGULATION_OF_CHEMOTAXIS | 48 | 1.9 | 0.02575 |
| | GO_POSITIVE_REGULATION_OF_LYMPHOCYTE_MIGRATION | 25 | 1.86 | 0.03391 |
| | GO_REGULATION_OF_LYMPHOCYTE_CHEMOTAXIS | 18 | 1.86 | 0.03401 |
| | GO_NEGATIVE_REGULATION_OF_LEUKOCYTE_MIGRATION | 31 | 1.86 | 0.03553 |
| **Interferon gamma response** | GO_CELLULAR_RESPONSE_TO_INTERFERON_GAMMA | 97 | 2.49 | 0 |
| | GO_RESPONSE_TO_INTERFERON_GAMMA | 115 | 2.41 | 0 |
| | GO_INTERFERON_GAMMA_MEDIATED_SIGNALING_PATHWAY | 56 | 2.02 | 0.00867 |
| **Toll Like Receptors (TLRs)** | GO_TOLL_LIKE_RECEPTOR_SIGNALING_PATHWAY | 78 | 2.46 | 0 |
| | GO_REGULATION_OF_TOLL_LIKE_RECEPTOR_SIGNALING_PATHWAY | 43 | 2.12 | 0.00266 |
| | GO_TOLL_LIKE_RECEPTOR_4_SIGNALING_PATHWAY | 16 | 1.99 | 0.01138 |
| | GO_NEGATIVE_REGULATION_OF_TOLL_LIKE_RECEPTOR_SIGNALING_PATHWAY | 23 | 1.96 | 0.01548 |
| | GO_MYD88_DEPENDENT_TOLL_LIKE_RECEPTOR_SIGNALING_PATHWAY | 30 | 1.86 | 0.03518 |
| **TNFalpha** | GO_POSITIVE_REGULATION_OF_TUMOR_NECROSIS_FACTOR_SUPERFAMILY_CYTOKINE_PRODUCTION | 54 | 2.45 | 0 |
| | GO_REGULATION_OF_TUMOR_NECROSIS_FACTOR_SUPERFAMILY_CYTOKINE_PRODUCTION | 93 | 2.4 | 0 |
| | GO_NEGATIVE_REGULATION_OF_TUMOR_NECROSIS_FACTOR_SUPERFAMILY_CYTOKINE_PRODUCTION | 39 | 1.96 | 0.01479 |
| | GO_REGULATION_OF_TUMOR_NECROSIS_FACTOR_BIOSYNTHETIC_PROCESS | 15 | 1.81 | 0.04811 |
| **Inflammatory response** | GO_INFLAMMATORY_RESPONSE | 397 | 2.46 | 0 |
| | GO_POSITIVE_REGULATION_OF_INFLAMMATORY_RESPONSE | 98 | 2.38 | 0 |
| | GO_REGULATION_OF_INFLAMMATORY_RESPONSE | 266 | 2.21 | 0.00076 |
| | GO_REGULATION_OF_ACUTE_INFLAMMATORY_RESPONSE | 66 | 1.94 | 0.01855 |
| **Chemokine signaling** | GO_CHEMOKINE_RECEPTOR_BINDING | 48 | 2.41 | 0 |
| | GO_CHEMOKINE_ACTIVITY | 41 | 2.4 | 0 |
| | GO_CHEMOKINE_MEDIATED_SIGNALING_PATHWAY | 62 | 2.4 | 0 |
| | GO_CCR_CHEMOKINE_RECEPTOR_BINDING | 27 | 2.29 | 0.00034 |
| | GO_CXCR_CHEMOKINE_RECEPTOR_BINDING | 15 | 2.08 | 0.00426 |

| | | | | |
|---|---|---|---|---|
| | GO_POSITIVE_REGULATION_OF_CHEMOKINE_PRODUCTION | 47 | 2.04 | 0.00706 |
| | GO_REGULATION_OF_CHEMOKINE_PRODUCTION | 62 | 1.95 | 0.01722 |
| **Regulation of endocytosis** | GO_RECEPTOR_MEDIATED_ENDOCYTOSIS | 196 | 2.35 | 0 |
| | GO_REGULATION_OF_ENDOCYTOSIS | 181 | 2.22 | 0.00076 |
| | GO_ENDOCYTOSIS | 437 | 2.21 | 0.00081 |
| | GO_POSITIVE_REGULATION_OF_ENDOCYTOSIS | 104 | 2.16 | 0.00151 |
| **Extracellular matrix disassembly** | GO_EXTRACELLULAR_MATRIX_DISASSEMBLY | 68 | 2.4 | 0 |
| | GO_EXTRACELLULAR_MATRIX_BINDING | 48 | 2.01 | 0.00936 |
| **IL6 synthesis** | GO_REGULATION_OF_INTERLEUKIN_6_PRODUCTION | 96 | 2.17 | 0.00137 |
| | GO_POSITIVE_REGULATION_OF_INTERLEUKIN_6_PRODUCTION | 63 | 2.14 | 0.00196 |
| **VEGF-VEGFR pathway** | GO_REGULATION_OF_VASCULAR_ENDOTHELIAL_GROWTH_FACTOR_PRODUCTION | 29 | 2.12 | 0.00259 |
| | GO_VASCULAR_ENDOTHELIAL_GROWTH_FACTOR_RECEPTOR_SIGNALING_PATHWAY | 65 | 1.84 | 0.03939 |
| | GO_POSITIVE_REGULATION_OF_VASCULAR_ENDOTHELIAL_GROWTH_FACTOR_PRODUCTION | 25 | 1.83 | 0.04149 |
| **Integrin-mediated cell adhesion** | GO_INTEGRIN_MEDIATED_SIGNALING_PATHWAY | 73 | 2.08 | 0.00432 |
| | GO_INTEGRIN_BINDING | 96 | 2.05 | 0.00646 |
| **Antigen processing and presentation** | GO_ANTIGEN_PROCESSING_AND_PRESENTATION_OF_PEPTIDE_OR_POLYSACCHARIDE_ANTIGEN_VIA_MHC_CLASS_II | 77 | 2.1 | 0.00348 |
| | GO_ANTIGEN_PROCESSING_AND_PRESENTATION_OF_PEPTIDE_ANTIGEN_VIA_MHC_CLASS_II | 77 | 2.08 | 0.00431 |
| | GO_ANTIGEN_BINDING | 67 | 1.99 | 0.01098 |
| | GO_ANTIGEN_PROCESSING_AND_PRESENTATION_OF_PEPTIDE_ANTIGEN | 146 | 1.89 | 0.02782 |
| | GO_ANTIGEN_PROCESSING_AND_PRESENTATION | 180 | 1.89 | 0.02819 |
| **Angiogenesis-vasculature development** | GO_POSITIVE_REGULATION_OF_VASCULATURE_DEVELOPMENT | 124 | 1.93 | 0.01933 |
| | GO_ANGIOGENESIS | 276 | 1.82 | 0.04686 |

## ONCOGENIC GENE SETS (C6)

| Biological process | Gene set name | Size | NES | FDR q-val |
|---|---|---|---|---|
| **KRAS** | KRAS.LUNG.BREAST_UP.V1_UP | 127 | 2.26 | 0 |
| | KRAS.50_UP.V1_UP | 45 | 2.09 | 0.00015 |
| | KRAS.KIDNEY_UP.V1_UP | 133 | 2.09 | 0.00013 |
| | KRAS.300_UP.V1_UP | 133 | 2.09 | 0.00012 |
| | KRAS.600.LUNG.BREAST_UP.V1_UP | 248 | 1.91 | 0.00143 |
| | KRAS.600_UP.V1_UP | 253 | 1.86 | 0.00235 |
| | KRAS.KIDNEY_UP.V1_DN | 121 | 1.8 | 0.00469 |
| | KRAS.BREAST_UP.V1_UP | 126 | 1.73 | 0.00864 |
| | KRAS.LUNG_UP.V1_UP | 123 | 1.71 | 0.009 |
| | KRAS.DF.V1_UP | 160 | 1.52 | 0.04356 |

**REFERENCES**


1. Garcia-Rodríguez L, Abate-Daga D, Rojas A, González JR, Fillat C. E-cadherin contributes to the bystander effect of TK/GCV suicide therapy and enhances its antitumoral activity in pancreatic cancer models. *Gene Ther.* 2011;18(1):73–81.
2. Newman AM, Liu CL, Green MR, et al. Robust enumeration of cell subsets from tissue expression profiles. *Nat. Methods*. 2015;12(5):453–457.
3. Kleinman HK, Cid MC. Preparation of endothelial cells. *Curr. Protoc. cell Biol.* 2001;Chapter 2(1):Unit 2.3.
4. Carreras J, Lopez-Guillermo A, Kikuti YY, et al. High TNFRSF14 and low BTLA are associated with poor prognosis in Follicular Lymphoma and in Diffuse Large B-cell Lymphoma transformation. *J. Clin. Exp. Hematop.* 2019;59(1):1–16.
